# Design, Synthesis and Evaluation of Oxazaborine Inhibitors of the NLRP3 Inflammasome

**DOI:** 10.1002/cmdc.201700731

**Published:** 2018-02-05

**Authors:** Alex G. Baldwin, Victor S. Tapia, Tessa Swanton, Claire S. White, James A. Beswick, David Brough, Sally Freeman

**Affiliations:** ^1^ Division of Pharmacy and Optometry, School of Health Sciences, Faculty of Biology, Medicine and Health, Manchester Academic Health Science Centre The University of Manchester Stopford Building, Oxford Road Manchester M13 9PT UK; ^2^ Division of Neuroscience and Experimental Psychology, School of Biological Sciences, Faculty of Biology, Medicine and Health, Manchester Academic Health Science Centre The University of Manchester AV Hill Building, Oxford Road Manchester M13 9PT UK

**Keywords:** boron, inflammation, NLRP3 inflammasome, oxazaborines, structure–activity relationships

## Abstract

The NLRP3 inflammasome is an important regulator of the sterile inflammatory response, and its activation by host‐derived sterile molecules leads to the intracellular activation of caspase‐1, processing of the pro‐inflammatory cytokines interleukin‐1β (IL‐1β)/IL‐18, and pyroptotic cell death. Inappropriate activation of NLRP3 drives a chronic inflammatory response and is implicated in several non‐communicable diseases, including gout, atherosclerosis, type II diabetes and Alzheimer's disease. In this study, we report the design, synthesis and biological evaluation of novel boron compounds (NBCs) as NLRP3 inflammasome inhibitors. Structure–activity relationships (SAR) show that 4‐fluoro substituents on the phenyl rings retain NLRP3 inhibitory activity, whereas more steric and lipophilic substituents diminish activity. Loss of inhibitory activity is also observed if the CCl_3_ group on the oxazaborine ring is replaced by a CF_3_ group. These findings provide additional understanding of the NBC series and will aid in the development of these NLRP3 inhibitors as tool compounds or therapeutic candidates for sterile inflammatory diseases.

## Introduction

Sterile inflammation is a host‐driven immune response to injury in the absence of infection.^1^ A central regulator of the sterile inflammatory response is the NOD‐like receptor, pyrin domain‐containing protein 3 (NLRP3), a soluble pattern recognition receptor (PRR) whose activation facilitates release of the pro‐inflammatory cytokines interleukin‐1β (IL‐1β) and IL‐18 by forming a multiprotein complex called the NLRP3 inflammasome.^2^, ^3^ NLRP3 is activated by various stimuli, including host‐derived endogenous molecules released by necrosis termed damage‐associated molecular patterns (DAMPs).^2^, ^3^ Given the structural diversity of its known agonists, it is unlikely that known DAMPs engage NLRP3 directly^4^ and there have been several proposed mechanisms for NLRP3 activation which converge on a two‐step signalling process.^5^, ^6^ The first step is referred to as priming, and an initial stimulus (e.g., a TLR ligand) is required to upregulate the intracellular levels of NLRP3 and pro‐IL‐1β. The second step is termed activation in which the primed cell encounters a second stimulus (e.g., a NLRP3 activating DAMP) that leads to NLRP3 inflammasome formation. This process is ATP‐dependent and requires the association of NLRP3 with the adaptor protein, apoptosis‐associated speck‐like protein containing a CARD (ASC). The inactive zymogen pro‐caspase‐1 is then recruited to the NLRP3 inflammasome via CARD–CARD homotypic interactions with ASC, resulting in its proximity‐induced autocleavage into active caspase‐1. Caspase‐1 then cleaves pro‐IL‐1β/IL‐18 into their biologically active mature forms IL‐1β/IL‐18 which are subsequently released from the cell into the extracellular space where they drive an inflammatory response.^4^, ^5^, ^6^, ^7^ Activation of caspase‐1 also leads to a form of cell death termed pyroptosis.^4^

IL‐1β and NLRP3 activation are well characterised in a number of non‐communicable diseases involving sterile inflammation including gout,^8^ atherosclerosis^9^ and type II diabetes (T2D).^10^, ^11^ Neuroinflammation caused by microglial activation is also often dependent on NLRP3 and IL‐1β and is associated with depression^12^ and Alzheimer's disease (AD).^13^, ^14^ Gain‐of‐function mutations in the *NLRP3* gene causes spontaneous IL‐1β release in patients with cryopyrin‐associated periodic syndrome (CAPS) diseases that are characterised by fever, rashes and extensive joint pain.^15^

Given the critical role of NLRP3 and IL‐1β in human disease,^16^ there has been great interest in the development of pharmacological agents that target the NLRP3‐IL‐1β axis. Although anti‐IL‐1β therapy using the biological IL‐1β inhibitors rilonacept (Arcalyst), canakinumab (Ilaris) and anakinra (Kineret) are highly effective and are currently used clinically, blockade of NLRP3 inflammasome activation would offer distinct advantages. Firstly, biological IL‐1β inhibitors are only able to target IL‐1β whereas small molecule NLRP3 inhibitors are likely to inhibit both IL‐1β and IL‐18 release, block pyroptosis,^17^ and prevent the secretion of inflammasome components that are themselves pro‐inflammatory.^18^, ^19^ Secondly, biological IL‐1β inhibitors are protein‐based therapeutics that are expensive, with anakinra requiring high dosages and frequent administration.^20^ Additionally, they are unlikely to cross the blood–brain barrier (BBB) easily and thus are limited to peripheral inflammatory diseases. Therefore it would be preferable to develop small molecule therapeutics capable of blocking NLRP3 inflammasome activation as they could be of use for CNS indications, are able to be administered orally and are likely to be more cost‐effective alternatives.

A number of small molecule inhibitors of the NLRP3 inflammasome have been previously described.^21^ However, many of the reported small molecule NLRP3 inhibitors have potency in the micromolar range, show poor selectivity or contain reactive functional groups, limiting their development as potential drug candidates. A notable exception is MCC950 (formerly known as CRID3 or CP‐456,773), the most potent and selective inhibitor of the NLRP3 inflammasome to date,^22^ and its hybrids with known sulfonylurea drugs are being developed as dual action insulin secretagogues and NLRP3 inhibitors for T2D.^23^ There is also commercial interest in the development of sulfonylurea drugs as NLRP3 inflammasome inhibitors, with recent patents in the sulfonylurea space highlighting the significant current interest in the NLRP3 inhibitor area.^24^, ^25^ Nevertheless, there is still a need for new NLRP3 inhibitors as there are currently no approved small molecule inhibitors of the NLRP3 inflammasome available clinically.

**Figure 1:**
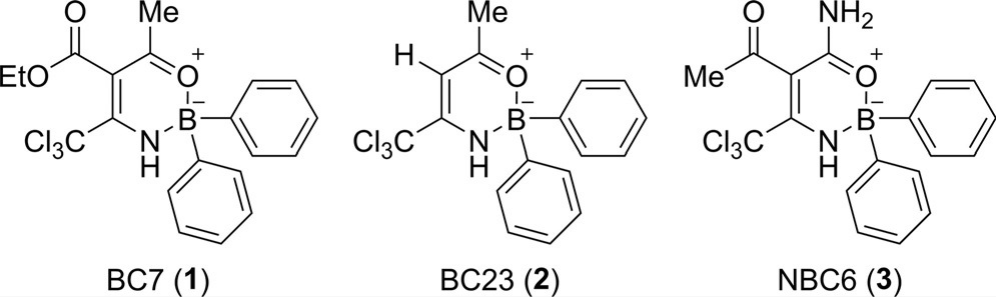
Boron‐based NLRP3 inflammasome inhibitors.

We recently reported on the discovery of new boron‐based small molecules as potent NLRP3 inhibitors.^26^ Three of the oxazaborine compounds screened, BC7 (**1**), BC23 (**2**) and NBC6 (**3**, Figure 1) were particularly effective inhibitors of IL‐1β release. The pharmacophore for these molecules responsible for NLRP3 inhibition is the oxazaborine ring and the highly electron‐withdrawing trichloromethyl (CCl_3_) group. However, the impact of phenyl ring substitutions on IL‐1β release was not assessed. Additionally, the presence of the CCl_3_ group significantly contributes to the high lipophilicity of these oxazaborine inhibitors, limiting their drug‐likeness.

Here we explored structure–activity relationships (SAR) of phenyl ring substitutions based on the known NLRP3 inhibitors BC23 (**2**) and NBC6 (**3**), in addition to seeking alternative bioisosteres of the CCl_3_ group in the search for new NLRP3 inhibitors with improved potency and drug‐like properties.

## Results and Discussion

### Chemistry

**Scheme 1:**
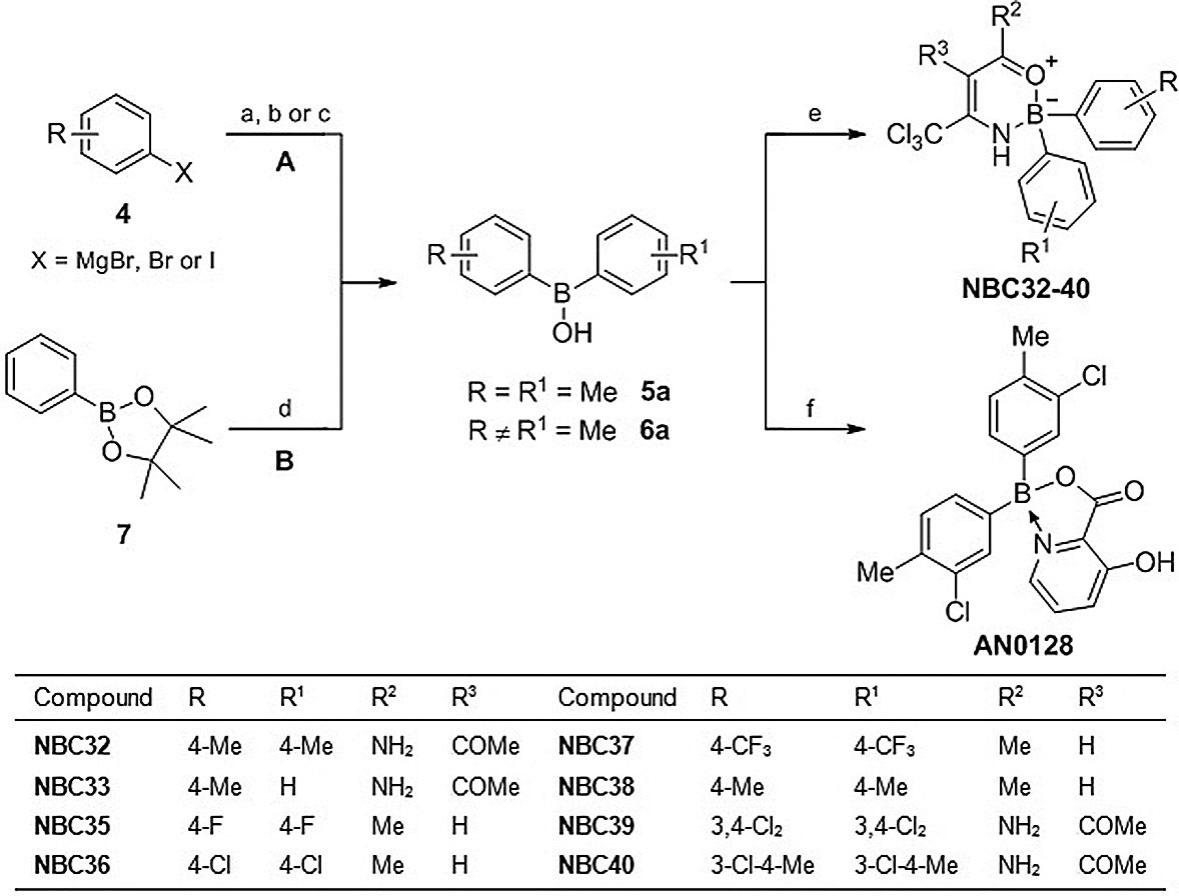
Synthesis of 2,2‐diaryl‐1,3,2‐oxazaborines (NBC32‐40) and AN0128: a) B(OMe)_3_, THF, RT, 3 h (for X=MgBr); b) Mg, I_2_, B(OMe)_3_, THF, 40 °C, 3 h (for X=Br); c) *i*PrMgCl, B(OMe)_3_, THF, 0 °C→RT, 16 h (for X=I); d) **4** (X=MgBr), THF, RT, 3 h; e) (*Z*)‐4‐amino‐5,5,5‐trichloropent‐3‐en‐2‐one or (*Z*)‐2‐acetyl‐3‐amino‐4,4,4‐trichlorobut‐2‐enamide, THF, 50 °C, 16 h; f) 3‐hydroxypicolinic acid, EtOH, reflux, 15 min.

Using the Topliss scheme for aromatic substituents,^27^ a series of mono‐ and disubstituted oxazaborine novel boron compounds (NBCs) were synthesised by considering both lipophilicity and electronic factors in order to determine the optimal substituent as efficiently as possible. Borinic acids with identical substituted phenyl rings (Scheme 1, Method A) were first synthesised by reacting two molar equivalents of an aryl halide (**4**, X=MgBr, Br or I) with magnesium turnings or isopropylmagnesium chloride (*i*PrMgCl), followed by treatment with one molar equivalent of trimethyl borate (B(OMe)_3_) to afford symmetrical borinic acids (**5**).^28^ Alternatively, monosubstituted aryl(phenyl)borinic acids (Scheme 1, Method B) were synthesised by treating **4** (when X=MgBr), such as *p*‐tolylmagnesium bromide, with a stoichiometric quantity of phenylboronic acid pinacol ester (**7**) to give the asymmetric borinic acid (**6**).

The synthesised borinic acids **5** and **6** were then reacted with either (*Z*)‐2‐acetyl‐3‐amino‐4,4,4‐trichlorobut‐2‐enamide or (*Z*)‐4‐amino‐5,5,5‐trichloropent‐3‐en‐2‐one^26^ at 50 °C in THF to give substituted NBC6 (NBC32‐33 and NBC40) or BC23 analogues (NBC35‐39), respectively (Scheme 1).

AN0128, a known borinic acid picolinate ester prepared by Anacor Pharmaceuticals, was also synthesised in a two‐step method according to the reported procedure (Scheme 1, Method A).^29^ The rationale for the synthesis of AN0128 was due to its structural similarity with our oxazaborine inhibitors, in addition to its known potent antibacterial and anti‐inflammatory activities. AN0128 showed 99 % inhibition of IL‐1β release and 100 % inhibition of TNF‐α release from human LPS‐induced PBMCs at a concentration of 10 μm,^29^ and has since entered phase II clinical trials for the treatment of atopic dermatitis, acne and periodontal disease.^30^ Therefore it was of interest to compare the inhibitory activity of the oxazaborine derivatives with AN0128 in the IL‐1β release assay.

Although the synthesis of oxazaborine derivatives containing electron‐donating substituents (R=R^1^=4‐OMe, 4‐NMe_2_, Scheme 1) were attempted, we found that the bromine‐magnesium (Br‐Mg) exchange reaction of aryl bromides with B(OMe)_3_ was highly dependent on aromatic ring substitution. Electron‐withdrawing aryl halides (R=R^1^=4‐F, 4‐Cl, 4‐CF_3_, 3,4‐Cl_2_, Scheme 1) generally reacted well with magnesium turnings to give the corresponding borinic acids after aqueous work‐up. In contrast, electron‐donating substituents (R=R^1^=4‐CH_3_, 4‐OMe, 4‐NMe_2_, Scheme 1) caused the aryl bromide to react very slowly with magnesium turnings and, for 4‐bromoanisole and 4‐bromo‐*N*,*N*‐dimethylaniline, led to a number of side‐products. The lack of significant reactivity observed for electron‐rich aryl bromides toward Br‐Mg exchange is in line with previous reports.^31^, ^32^

**Scheme 2:**
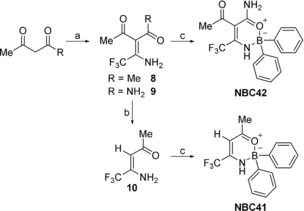
Synthesis of trifluoromethyl derivatives of BC23 (NBC41) and NBC6 (NBC42): a) CF_3_CN, Zn(acac)_2_, CH_2_Cl_2_, RT, 18 h; b) K_2_CO_3_, EtOH, 50 °C, 24 h (for R=Me); c) DPBA, THF, 50 °C, 16 h.

Analogues of BC23 (**2**) and NBC6 (**3**) were then designed where the CCl_3_ group was replaced with the bioisosteric trifluoromethyl (CF_3_) group (Scheme 2). We had previously shown that only electron‐withdrawing substituents at this position were effective NLRP3 inhibitors,^26^ thus the CF_3_ moiety should retain similar electron‐withdrawing properties to that of a CCl_3_ group but is significantly less lipophilic. Additionally, replacement of the polychlorinated centre would rule out its potentially labile nature with regards to attack by nucleophiles or radicals.

CF_3_CN was prepared from trifluoroacetamide using trifluoroacetic anhydride according to the method described by Parker.^33^ CF_3_CN was reacted with either acetylacetone or acetoacetamide to give **8** and **9**, respectively (Scheme 2). Intermediate **8** was deacetylated under basic conditions using a saturated solution of K_2_CO_3_ in EtOH to give deacetylated β‐trifluoroenaminone **10**.^34^ Subsequently, intermediates **9** and **10** were borylated with diphenylborinic anhydride (DPBA) to give analogues NBC41 and NBC42 (Scheme 2).

### Inhibition of IL‐1β release

**Figure 2:**
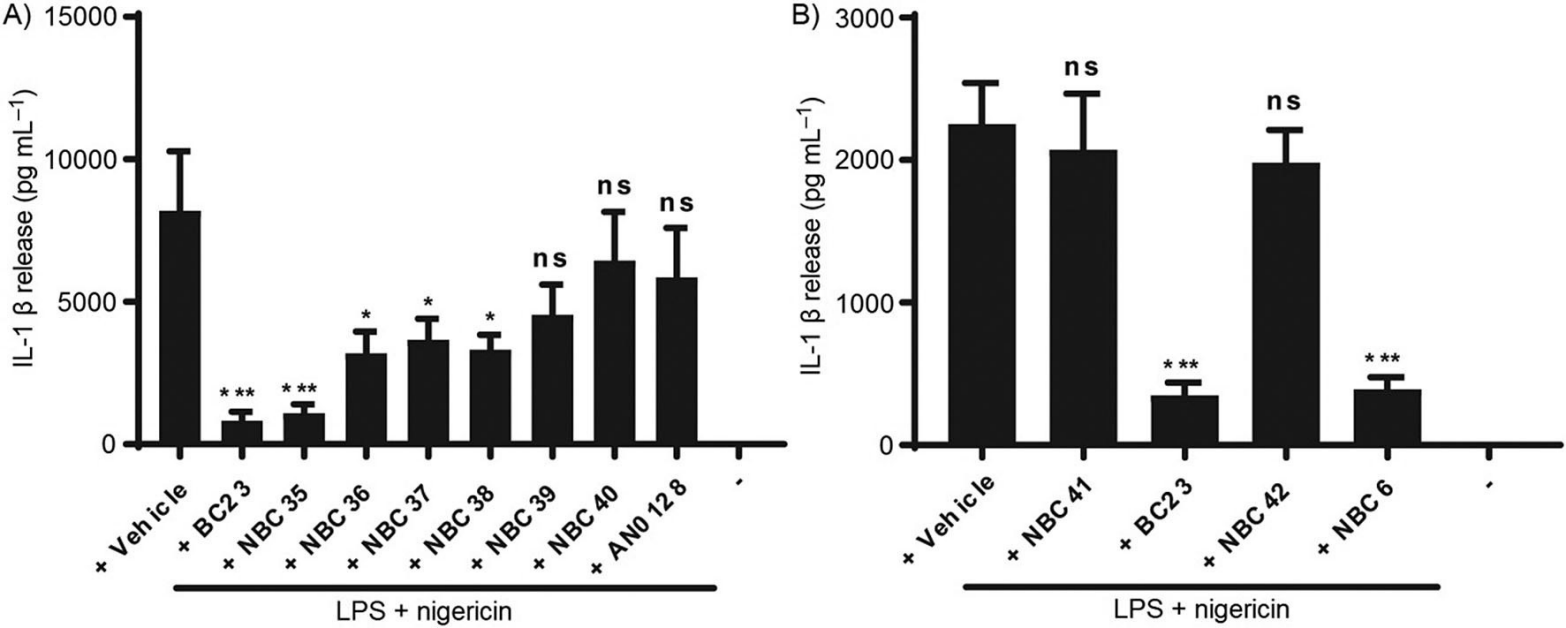
Effects of the new NBCs on IL‐1β release. iBMDMs were treated with LPS (1 μg mL^−1^, 4 h) followed by vehicle or drugs with ring substitutions (BC23 vs. NBC35‐40, AN0128) (A), or drugs with CCl_3_ substituted for CF_3_ (BC23 vs. NBC41; NBC6 vs. NBC42) (B). Drugs were incubated at 10 μm 15 min before stimulation with nigericin (10 μm, 1 h). IL‐1β was measured by ELISA. Data are the mean±SEM with a minimum of three experiments per group; ns=non‐significant, *p<0.05, ***p<0.001 vs. vehicle.

We initially tested the effect of modifying the phenyl rings of NBCs on their ability to inhibit NLRP3 inflammasome‐dependent release of IL‐1β from macrophages. Immortalised bone marrow derived macrophages (iBMDMs) were treated with LPS (1 μg mL^−1^, 4 h) to prime the cells and induce expression of pro‐IL‐1β. The primed cells were then incubated with vehicle (0.5 % DMSO) or inhibitor (10 μm) for 15 minutes before the NLRP3 inflammasome activator nigericin (10 μm, 1 h) was added to cells. Supernatants were removed and IL‐1β levels were analysed by ELISA. Nigericin induced release of IL‐1β and this was inhibited by our parent molecule BC23 as expected (Figure 2 A). Addition of a small electron withdrawing fluorine at the *para* position of each phenyl ring (NBC35) had minimal effect on inhibitory activity (Figure 2 A). However, inhibitory activity was decreased when bulkier, more lipophilic substituents were added (Figure 2 A, NBC36‐40). The electron‐withdrawing properties of aryl substituents were insignificant for inhibitory activity as the bioisosteric 4‐Cl (NBC36), 4‐CF_3_ (NBC37) and 4‐CH_3_ (NBC38) derivatives all inhibited IL‐1β release to a similar extent. This observation is further supported by the significantly decreased activities of 3,4‐Cl_2_ (NBC39) and 3‐Cl,4‐Me (NBC40) derivatives which possess additional substitutions around the phenyl rings. In contrast, there was an observed correlation between increasing lipophilicity and decreasing inhibitory activity across the BC23 series (NBC35 < NBC36 ≈NBC37 ≈NBC38 < NBC39). These observations were also in agreement across the NBC6 series, where the disubstituted‐*p*‐tolyl analogue NBC32 had slightly lower IL‐1β inhibitory activity than the monosubstituted‐*p*‐tolyl analogue NBC33 and parent compound NBC6 in THP‐1 cells (Figure S1). These results suggest that aryl ring substitution, particularly with more steric and lipophilic substituents, is unlikely to enhance the activity of the NBCs.

It was surprising to note the lack of inhibitory activity of AN0128 in the IL‐1β release assay given its known anti‐inflammatory effects against IL‐1β and TNF‐α.^29^ This could be due to differences in cell type (iBMDMs vs. PBMCs) or the two‐step priming and activating stimuli used in this study relative to a single LPS stimulus used previously.^29^ Nevertheless, the observation that AN0128 had little effect on IL‐1β release in our NLRP3 inflammasome‐dependent assay is an important finding and suggests that the anti‐inflammatory action of AN0128 is independent of blocking NLRP3 inflammasome activation.

We then tested the effects of BC23 and NBC6 analogues where the CCl_3_ group had been replaced with the bioisosteric trifluoromethyl (CF_3_) group (Scheme 2). As we had previously reported that only electron‐withdrawing substituents at this position were effective NLRP3 inhibitors,^26^ we proposed that substituting CCl_3_ for a CF_3_ group would test the importance of lipophilicity whilst retaining similar electron‐withdrawing properties. Using the iBMDM model as described, nigericin induced a significant release of IL‐1β and this was inhibited by BC23 and NBC6 (Figure 2 B). However, both NBC41 (the CF_3_ analogue of BC23) and NBC42 (the CF_3_ analogue of NBC6) were ineffective under these conditions (Figure 2 B). These results, together with our previous observations, clearly demonstrate that the CCl_3_ group fulfils both the lipophilic and electron‐withdrawing properties required at this position that is critical for the inhibitory effects of the NBCs in the IL‐1β release assay (Figure 2 B).

Given that the CCl_3_ group is essential for IL‐1β inhibitory activity, preliminary chemical studies on BC23 to determine the potential lability of the CCl_3_ group to cysteine, amine‐based and oxygen‐based nucleophiles were performed. However, no evidence of CCl_3_ modification or loss was observed, except fragmentation under mass spectrometry conditions (data not shown). Therefore these initial studies suggest that the CCl_3_ group is not labile under these reaction conditions. It was noted that during these experiments, nucleophiles were found to attack the boron atom and undergo decomplexation. For example, BC23 cleanly hydrolyses into diphenylborinic acid and (*Z*)‐4‐amino‐5,5,5‐trichloropent‐3‐en‐2‐one in 9:1 [D_6_]DMSO/D_2_O solvent at 37 °C with a half‐life of ≈24 h, as monitored by ^1^H NMR spectroscopy (Figure S2).

## Conclusions

The NLRP3 inflammasome is a multi‐molecular protein complex that is critical for inflammatory responses. Its formation leads to activation of caspase‐1, which cleaves and activates IL‐1β.^5^ NLRP3 inflammasome activation is suggested to be important in diseases such as Alzheimer's disease,^13^ atherosclerosis,^9^ and metabolic disease such as type II diabetes.^10^ The importance of IL‐1β to disease was further highlighted following the recent publication of the CANTOS trial, where patients with a history of myocardial infarction were treated with canakinumab, a monoclonal antibody targeting IL‐1β.^35^ It was found that canakinumab treatment decreased the rate of recurrent cardiovascular events, and cancer mortality, in addition to many other clinical outcomes.^35^ The CANTOS findings have since led to several pharmaceutical companies seeking to develop molecules that target NLRP3 directly.^36^ There is thus a growing interest in inhibitors of the NLRP3 inflammasome.^21^

We recently published the NBC series of NLRP3 inflammasome inhibitors reporting that key features required for bioactivity were the oxazaborine ring and CCl_3_ group.^26^ Here we have further refined the SAR and shown that substitutions on the aryl rings do not enhance inhibitory activity, and that the lipophilicity of the CCl_3_ group is key to inhibitory activity. These discoveries provide new insight into the activity of the NBC series and will aid future development of the NBC molecules as inflammasome inhibitors.

## Experimental Section

### Chemistry

**General**: (*Z*)‐4‐Amino‐5,5,5‐trichloropent‐3‐en‐2‐one and (*Z*)‐2‐acetyl‐3‐amino‐4,4,4‐trichlorobut‐2‐enamide intermediates were prepared as previously described.^26^ All other chemicals, solvents and deuterated solvents were purchased from Sigma–Aldrich, Alfa‐Aesar or Fisher Scientific. ^1^H, ^13^C, ^11^B and ^19^F NMR spectra were recorded on a Bruker Avance 400 or 300 MHz spectrometer. Chemical shifts (*δ*) are defined in parts per million (ppm). ^1^H NMR spectra were referenced to tetramethylsilane (TMS, *δ*=0.0 ppm) or residual undeuterated solvent (CDCl_3_, *δ*=7.26 ppm; [D_6_]DMSO, *δ*=2.50 ppm). ^13^C NMR spectra were referenced to residual undeuterated solvent (CDCl_3_, *δ*=77.16 ppm, [D_6_]DMSO=39.52 ppm) as an internal reference. ^11^B NMR chemical shifts were referenced to external reference BF_3_⋅OEt_2_ (*δ*=0.0 ppm). ^19^F NMR chemical shifts were referenced using the deuterium lock signal of the solvent. ESI and APCI mass spectrometry was carried out on a Waters Acquity UPLC system connected to a Waters SQD2 mass spectrometer. Accurate mass determination was carried out on a Thermo Exactive Plus EMR Orbitrap LC–MS system. Molecular ion peaks are defined as mass/charge (*m*/*z*) ratios. Infrared spectroscopy was recorded on a JASCO FT/IR‐4100 spectrophotometer using the Spectra Manager II (JASCO) software package. Melting points were measuring using a Stuart SMP10 melting point apparatus. Lyophilisation was carried out using a Christ alpha1‐4 plus freeze dryer equipped with an Edwards vacuum pump. Microwave irradiation was carried out on a Biotage Initiator Classic microwave using 2–5 mL Biotage glass vials. Analytical thin‐layer chromatography (TLC) was performed using silica gel 60 on aluminium sheets coated with F254 indicator. All spots were visualised with KMnO_4_ or ultraviolet light using a MV Mineralight lamp (254/365) UVGL‐58. Flash column chromatography was performed using silica gel with particle size 40–63 μm. Evaporation of solvents was conducted on a Büchi Rotavapor R‐200.

**Di‐*p*‐tolylborinic acid (5 a)**: To an oven‐dried Schlenk flask under N_2_ was added *p*‐tolylmagnesium bromide (1.0 m in THF, 9.98 mL, 10 mmol) in anhydrous THF (5 mL). B(OMe)_3_ (0.55 mL, 5 mmol) was added dropwise to the reaction mixture and stirred at room temperature for 3 h. HCl (1 m, 10 mL) was then added and stirred for 30 min to quench the reaction. The reaction mixture was extracted with EtOAc (3×10 mL), washed with brine (1×10 mL), dried over MgSO_4_, filtered and evaporated in vacuo. The crude product was then purified by flash column chromatography (EtOAc/*n*‐hexane, 1:19) to give **5 a** as a colourless oil (0.61 g, 58 %). ^1^H NMR (300 MHz, CDCl_3_): *δ*=7.63 (d, *J*=7.8 Hz, 4 H, B‐Ar(*m*)), 7.18 (d, *J*=7.5 Hz, 4 H, B‐Ar(*o*)), 5.65 (br s, 1 H, OH), 2.33 ppm (s, 6 H, CH_3×_2); ^13^C NMR (75 MHz, CDCl_3_): *δ*=141.2 (B‐Ar(*p*)), 134.8 (B‐Ar(*o*)), 128.7 (B‐Ar(*m*)), 21.7 ppm (CH_3_), B‐Ar(*i*) quaternary signal not observed.

**5‐Acetyl‐6‐amino‐2,2‐bis(*p*‐tolyl)‐4‐(trichloromethyl)‐2,3‐dihydro‐1,3,2‐oxazaborinin‐1‐ium‐2‐uide (NBC32)**: (*Z*)‐2‐Acetyl‐3‐amino‐4,4,4‐trichlorobut‐2‐enamide (1.46 g, 5.95 mmol) was added to a solution of **5 a** (0.50 g, 2.38 mmol) in anhydrous THF (5 mL). The mixture was stirred at 50 °C under N_2_ for 16 h. The mixture was concentrated in vacuo and purified by flash column chromatography (EtOAc/*n*‐hexane, 2:23). The collected fractions were combined, evaporated in vacuo and stirred in cold *n*‐hexane (15 mL) for 30 min. The precipitate was then filtered and dried under vacuum to give NBC32 as a yellow solid (0.14 g, 14 %); mp: 145–148 °C (dec); ^1^H NMR (300 MHz, CDCl_3_): *δ*=9.23 (br s, 1 H, CONH_2_), 7.58 (br s, 1 H, Cl_3_C(NH)C=C), 7.29 (d, *J*=7.5 Hz, 4 H, B‐Ar(*o*)), 7.12 (d, 7.5 Hz, 4 H, B‐Ar(*m*)), 5.92 (br s, 1 H, CONH_2_), 2.34 (s, 6 H, CH_3×_2), 2.32 ppm (s, 3 H, CH_3_CO); ^13^C NMR (75 MHz, CDCl_3_): *δ*=198.1 (CH_3_ *C*O), 169.2 (CONH_2_), 165.5 (Cl_3_C(NH)*C*=*C*), 156.1 (B‐Ar(*i*)), 136.2 (B‐Ar(*p*)), 132.0 (B‐Ar(*o*)), 128.2 (B‐Ar(*m*)), 97.9 (Cl_3_C(NH)C=C), 94.5 (CCl_3_), 34.0 (*C*H_3_CO), 21.3 ppm (CH_3_); IR (neat): $\tilde{\nu }$ =3388 (N−H), 3313 (N−H), 1644 (C=O), 1608 (C=C, conjugated), 1561 cm^−1^ (C=C‐NH); MS(ES^−^) (*m*/*z*): 434.2 [*M*−H, ^10^B, ^35^Cl, ^35^Cl, ^35^Cl, 12 %]^−^, 435.2 [*M*−H, ^11^B, ^35^Cl, ^35^Cl, ^35^Cl, 100 %]^−^, 436.2 [*M*−H, ^10^B, ^35^Cl, ^35^Cl, ^37^Cl, 24 %]^−^, 437.2 [*M*−H, ^11^B, ^35^Cl, ^35^Cl, ^37^Cl, 50 %]^−^, 438.2 [*M*−H, ^10^B, ^35^Cl, ^37^Cl, ^37^Cl, 11 %]^−^, 439.2 [*M*−H, ^11^B, ^35^Cl, ^37^Cl, ^37^Cl, 10 %]^−^; MS(ES^+^) (*m*/*z*): 344.1 [*M*−tolyl, ^10^B, ^35^Cl, ^35^Cl, ^35^Cl, 10 %]^+^, 345.0 [*M*−tolyl, ^11^B, ^35^Cl, ^35^Cl, ^35^Cl, 27 %]^+^, 346.0 [*M*−tolyl, ^10^B, ^35^Cl, ^35^Cl, ^37^Cl, 12 %]^+^, 347.0 [*M*−tolyl, ^11^B, ^35^Cl, ^35^Cl, ^37^Cl, 36 %]^+^, 349.1 [*M*−tolyl, ^11^B, ^35^Cl, ^37^Cl, ^37^Cl, 21 %]^+^, 439.1 [*M*+H, ^11^B, ^35^Cl, ^35^Cl, ^37^Cl, 10 %]^+^; HRMS(ES^+^) (*m*/*z*): [*M*+H]^+^ calcd for C_20_H_21_^11^B^35^Cl_3_N_2_O_2_, 437.0756; found, 437.0738, error: 4.1 ppm.

**(Phenyl)(*p*‐tolyl)borinic acid (6 a)**: To an oven‐dried Schlenk flask under N_2_ was added *p*‐tolylmagnesium bromide (1.0 m in THF, 2.02 mL, 2.02 mmol) in anhydrous THF (5 mL). Phenylboronic acid pinacol ester (0.41 g, 2.00 mmol) in anhydrous THF (5 mL) was added dropwise to the reaction mixture and stirred at room temperature for 3 h. HCl (1 m, 10 mL) was then added and stirred for 30 min to quench the reaction. The reaction mixture was extracted with EtOAc (3×10 mL), washed with brine (1×10 mL), dried over MgSO_4_, filtered and evaporated in vacuo. The crude product was then purified by flash column chromatography (EtOAc/*n*‐hexane, 2:23) to give **6 a** as a colourless oil (0.18 g, 45 %). ^1^H NMR (300 MHz, CDCl_3_): *δ*=7.72 (d, *J*=7.8 Hz, 2 H, B‐Ph(*o*)), 7.64 (d, *J*=7.8 Hz, 2 H, B‐Ar(*o*)), 7.32–7.47 (m, 3 H, B‐Ph(*m*/*p*)), 7.19 (d, *J*=7.8 Hz, 2 H, B‐Ar(*m*)), 5.74 (br s, 1 H, OH), 2.33 ppm (s, 3 H, CH_3_); ^13^C NMR (75 MHz, CDCl_3_): *δ*=141.4 (B‐Ar(*p*)), 135.0 (B‐Ph(*o*)), 134.6 (B‐Ar(*o*)), 130.9 (B‐Ph(*p*)), 128.8 (B‐Ar(*m*)), 127.9 (B‐Ph(*m*)), 21.7 ppm (CH_3_), B‐Ar(*i*) and B‐Ph(*i*) quaternary signals not observed; MS(ES^−^) (*m*/*z*): 195.1 [*M*−H, ^11^B, 25 %]^−^; HRMS(ES^−^) (*m*/*z*): [*M*−H]^−^ calcd for C_13_H_12_^11^BO, 195.0987; found, 195.0973, error: 7.2 ppm.

**5‐Acetyl‐6‐amino‐2‐(phenyl)‐2‐(*p*‐tolyl)‐4‐(trichloromethyl)‐2,3‐dihydro‐1,3,2‐oxazaborinin‐1‐ium‐2‐uide (NBC33)**: (*Z*)‐2‐Acetyl‐3‐amino‐4,4,4‐trichlorobut‐2‐enamide (0.40 g, 1.65 mmol) was added to a solution of **6 a** (0.13 g, 0.66 mmol) in anhydrous THF (5 mL). The mixture was stirred at 50 °C under N_2_ for 16 h. The mixture was concentrated in vacuo and purified by flash column chromatography (EtOAc/*n*‐hexane, 2:23). The collected fractions were combined, evaporated in vacuo and stirred in cold *n*‐hexane (15 mL) for 30 min. The precipitate was then filtered and dried under vacuum to give NBC33 as a yellow solid (4.9 mg, 2 %); mp: 141–143 °C; ^1^H NMR (300 MHz, CDCl_3_): *δ*=9.25 (br s, 1 H, CONH_2_), 7.59 (br s, 1 H, Cl_3_C(NH)C=C), 7.40 (d, *J*=6.0 Hz, 2 H, B‐Ph(*o*)), 7.23–7.34 (m, 5 H, B‐Ph(*m*/*p*) & B‐Ar(*o*)), 7.12 (d, *J*=7.8 Hz, 2 H, B‐Ar(*m*)), 5.94 (br s, 1 H, CONH_2_), 2.34 (s, 3 H, CH_3_), 2.31 ppm (s, 3 H, CH_3_); ^13^C NMR (75 MHz, CDCl_3_): *δ*=192.8 (CH_3_ *C*O), 163.9 (Cl_3_C(NH)*C*=*C*), 160.3 (CONH_2_), 131.1 (B‐Ar(*p*)), 126.8 (B‐Ph(*o*)), 126.6 (B‐Ar(*o*)), 123.0 (B‐Ar(*m*)), 122.1 (B‐Ph(*m*)), 121.5 (B‐Ph(*p*)), 92.7 (Cl_3_C(NH)C=C), 89.2 (CCl_3_), 28.7 (*C*H_3_CO), 16.0 ppm (CH_3_), B‐Ar(*i*) and B‐Ph(*i*) quaternary signals not observed; MS(ES^−^) (*m*/*z*): 420.2 [*M*−H, ^10^B, ^35^Cl, ^35^Cl, ^35^Cl, 12 %]^−^, 421.1 [*M*−H, ^11^B, ^35^Cl, ^35^Cl, ^35^Cl, 80 %]^−^, 422.2 [*M*−H, ^10^B, ^35^Cl, ^35^Cl, ^37^Cl, 33 %]^−^, 423.2 [*M*−H, ^11^B, ^35^Cl, ^35^Cl, ^37^Cl, 100 %]^−^, 424.1 [*M*−H, ^10^B, ^35^Cl, ^37^Cl, ^37^Cl, 13 %]^−^, 425.1 [*M*−H, ^11^B, ^35^Cl, ^37^Cl, ^37^Cl, 24 %]^−^; HRMS(ES^+^) (*m*/*z*): [*M*+H]^+^ calcd for C_19_H_19_^11^B^35^Cl_3_N_2_O_2_, 423.0600; found, 423.0605, error: 1.2 ppm.

**General procedure for synthesis of 2,2‐bisaryl‐1,3,2‐oxazaborines**: Using an adapted procedure,^28^ to an oven‐dried Schlenk flask under N_2_ was added magnesium turnings (2.2 equiv), anhydrous THF (5 mL) and a small crystal of I_2_. The reaction was stirred at 40 °C for 30 min until complete decolourisation. A solution of aryl bromide (2 equiv) and B(OMe)_3_ (1 equiv) in anhydrous THF (5 mL) was then added dropwise to the reaction mixture and then stirred for an additional 3 h at 40 °C. After cooling to room temperature, 1 m HCl (10 mL) was added and stirred for 30 min to quench the reaction. The reaction mixture was extracted with EtOAc (3×10 mL), washed with brine (1×10 mL), dried over MgSO_4_, filtered and evaporated in vacuo to give the corresponding crude bisarylborinic acid, typically as a solid. (*Z*)‐4‐Amino‐5,5,5‐trichloropent‐3‐en‐2‐one (1.5 equiv) was then added to crude bisarylborinic acid (1 equiv) in anhydrous THF (5 mL). The reaction was stirred at 50 °C for 16 h under N_2_. The reaction mixture was concentrated and purified by flash column chromatography using the indicated solvent system. Collected fractions were evaporated in vacuo and stirred in the minimum amount of cold *n*‐hexane for 30 min to induce precipitation. The precipitate was then filtered and dried under vacuum to give the corresponding oxazaborine product. Percentage yields are reported over two steps.

**2,2‐Bis(4‐fluorophenyl)‐6‐methyl‐4‐(trichloromethyl)‐2,3‐dihydro‐1,3,2‐oxazaborinin‐1‐ium‐2‐uide (NBC35)**: EtOAc/*n*‐hexane, 1:9. Reaction scale: B(OMe)_3_ (0.56 mL, 5.00 mmol), 1‐bromo‐4‐fluorobenzene (1.10 mL, 10 mmol) and magnesium turnings (0.27 g, 11 mmol) gives bis(4‐fluorophenyl)borinic acid (1.20 g, quant). Bis(4‐fluorophenyl)borinic acid (1.20 g, 5.50 mmol) and (*Z*)‐4‐amino‐5,5,5‐trichloropent‐3‐en‐2‐one (0.89 g, 4.40 mmol) gives NBC35 as a yellow solid (0.22 g, 12 % over two steps); mp: 130–131 °C; ^1^H NMR (400 MHz, CDCl_3_): *δ*=7.31 (dd ≈t, ^3^ *J*_HH_ ≈^4^ *J*_HF_=6.6 Hz, 4 H, B‐Ar(*o*)), 7.00 (t, ^3^ *J*_HH_ ≈^3^ *J*_HF_=8.4 Hz, 4 H, B‐Ar(*m*)), 5.83 (s, 1 H, Cl_3_C(NH)C=C*H*), 2.25 ppm (s, 3 H, CH_3_CO), NH signal is overlapping with triplet at 7.31 ppm; ^13^C NMR (100 MHz, CDCl_3_): *δ*=186.5 (CH_3_ *C*O), 166.1 (Cl_3_C(NH)*C*=*C*H), 162.6 (d, ^1^ *J*_CF_=242.6 Hz, B‐Ar(*p*)), 133.4 (d, ^3^ *J*_CF_=27.6 Hz, B‐Ar(*o*)), 114.4 (d, ^2^ *J*_CF_=77.2 Hz, B‐Ar(*m*)), 92.8 (CCl_3_), 92.0 (Cl_3_C(NH)C=CH), 24.9 ppm (*C*H_3_CO), B‐Ar(*i*) quaternary signal not observed; ^11^B{^1^H} NMR (128 MHz, CDCl_3_): *δ*=4.11 ppm; ^19^F{^1^H} NMR (376 MHz, CDCl_3_): *δ*=−116.1 ppm; MS(ES^−^) (*m*/*z*): 399.1 [*M*−H, ^10^B, ^35^Cl, ^35^Cl, ^35^Cl, 14 %]^−^, 400.1 [*M*−H, ^11^B, ^35^Cl, ^35^Cl, ^35^Cl, 70 %]^−^, 401.1 [*M*−H, ^10^B, ^35^Cl, ^35^Cl, ^37^Cl, 40 %]^−^, 402.1 [*M*−H, ^11^B, ^35^Cl, ^35^Cl, ^37^Cl, 100 %]^−^, 403.1 [*M*−H, ^10^B, ^35^Cl, ^37^Cl, ^37^Cl, 15 %]^−^, 404.1 [*M*−H, ^11^B, ^35^Cl, ^37^Cl, ^37^Cl, 33 %]^−^; HRMS(ES^−^) (*m*/*z*): [*M*−H]^−^ calcd for C_17_H_12_^11^B^35^Cl_3_^19^F_2_NO, 400.0051; found, 400.0048, error: 0.7 ppm.

**2,2‐Bis(4‐chlorophenyl)‐6‐methyl‐4‐(trichloromethyl)‐2,3‐dihydro‐1,3,2‐oxazaborinin‐1‐ium‐2‐uide (NBC36)**: EtOAc/*n*‐hexane, 1:9. Reaction scale: B(OMe)_3_ (0.28 mL, 2.50 mmol), 1‐bromo‐4‐chlorobenzene (0.96 g, 5.00 mmol) and magnesium turnings (0.13 g, 5.50 mmol) gives bis(4‐chlorophenyl)borinic acid (0.71 g, 57 %). Bis(4‐chlorophenyl)borinic acid (0.71 g, 2.85 mmol) and (*Z*)‐4‐amino‐5,5,5‐trichloropent‐3‐en‐2‐one (0.86 g, 4.27 mmol) gives NBC36 as a yellow solid (0.38 g, 17 % over two steps); mp: 106–107 °C; ^1^H NMR (400 MHz, CDCl_3_): *δ*=7.25 (br s, 1 H, NH), 7.19 (br s, 8 H, B‐Ar(*o*/*m*)), 5.76 (s, 1 H, Cl_3_C(NH)C=C*H*), 2.18 ppm (s, 3 H, CH_3_CO); ^13^C NMR (100 MHz, CDCl_3_): *δ*=186.7 (CH_3_ *C*O), 166.3 (Cl_3_C(NH)*C*=*C*H), 133.2 (B‐Ar(*o*)), 133.1 (B‐Ar(*p*)), 127.8 (B‐Ar(*m*)), 92.7 (CCl_3_), 92.2 (Cl_3_C(NH)C=CH), 24.8 ppm (*C*H_3_CO), B‐Ar(*i*) quaternary signal not observed; ^11^B{^1^H} NMR (128 MHz, CDCl_3_): *δ*=3.82 ppm; MS(ES^−^) (*m*/*z*): 431.0 [*M*−H, ^10^B, ^35^Cl, ^35^Cl, ^35^Cl, ^35^Cl, ^35^Cl, 13 %]^−^, 432.0 [*M*−H, ^11^B, ^35^Cl, ^35^Cl, ^35^Cl, ^35^Cl, ^35^Cl, 75 %]^−^, 433.0 [*M*−H, ^10^B, ^35^Cl, ^35^Cl, ^35^Cl, ^35^Cl, ^37^Cl, 42 %]^−^, 434.0 [*M*−H, ^11^B, ^35^Cl, ^35^Cl, ^35^Cl, ^35^Cl, ^37^Cl, 100 %]^−^, 435.0 [*M*−H, ^10^B, ^35^Cl, ^35^Cl, ^35^Cl, ^37^Cl, ^37^Cl, 25 %]^−^, 436.0 [*M*−H, ^11^B, ^35^Cl, ^35^Cl, ^35^Cl, ^37^Cl, ^37^Cl, 88 %]^−^, 437.0 [*M*−H, ^10^B, ^35^Cl, ^35^Cl, ^37^Cl, ^37^Cl, ^37^Cl, 12 %]^−^, 438.0 [*M*−H, ^11^B, ^35^Cl, ^35^Cl, ^37^Cl, ^37^Cl, ^37^Cl, 30 %]^−^; HRMS(ES^−^) (*m*/*z*): [*M*−H]^−^ calcd for C_17_H_12_^11^B^35^Cl_5_NO, 431.9460; found, 431.9460, error: 0.0 ppm.

**6‐Methyl‐4‐(trichloromethyl)‐2,2‐bis(4‐(trifluoromethyl)phenyl)‐2,3‐dihydro‐1,3,2‐oxazaborinin‐1‐ium‐2‐uide (NBC37)**: EtOAc/*n*‐hexane, 1:19. Reaction scale: B(OMe)_3_ (0.28 mL, 2.50 mmol), 4‐bromobenzotrifluoride (0.70 mL, 5.00 mmol) and magnesium turnings (0.13 g, 5.50 mmol) gives bis(4‐trifluorophenyl)borinic acid (0.81 g, quant). Bis(4‐trifluorophenyl)borinic acid (0.81 g, 2.55 mmol) and (*Z*)‐4‐amino‐5,5,5‐trichloropent‐3‐en‐2‐one (0.77 g, 3.83 mmol) gives NBC37 as a yellow solid (0.43 g, 33 % over two steps); mp: 103–105 °C; ^1^H NMR (400 MHz, CDCl_3_): *δ*=7.56 (d, *J*=7.6 Hz, 4 H, B‐Ar(*m*)), 7.46 (d, *J*=7.6 Hz, 4 H, B‐Ar(*o*)), 7.41 (br s, 1 H, NH), 5.89 (s, 1 H, Cl_3_C(NH)C=C*H*), 2.30 ppm (s, 3 H, CH_3_CO); ^13^C NMR (100 MHz, CDCl_3_): *δ*=187.1 (CH_3_ *C*O), 166.7 (Cl_3_C(NH)*C*=*C*H), 131.8 (B‐Ar(*o*)), 129.2 (q, ^2^ *J*_CF_=31.3 Hz, B‐Ar(*p*)), 124.7 (q, ^1^ *J*_CF_=270.2 Hz, CF_3_), 124.4 (q, ^3^ *J*_CF_=3.6 Hz, B‐Ar(*m*)), 92.7 (Cl_3_C(NH)C=CH), 24.8 ppm (*C*H_3_CO), B‐Ar(*i*) and CCl_3_ quaternary signals not observed; ^11^B{^1^H} NMR (128 MHz, CDCl_3_): *δ*=3.56 ppm; ^19^F{^1^H} NMR (376 MHz, CDCl_3_): *δ*=−62.4 ppm; MS(ES^−^) (*m*/*z*): 499.1 [*M*−H, ^10^B, ^35^Cl, ^35^Cl, ^35^Cl, 22 %]^−^, 500.1 [*M*−H, ^11^B, ^35^Cl, ^35^Cl, ^35^Cl, 75 %]^−^, 501.1 [*M*−H, ^10^B, ^35^Cl, ^35^Cl, ^37^Cl, 45 %]^−^, 502.1 [*M*−H, ^11^B, ^35^Cl, ^35^Cl, ^37^Cl, 100 %]^−^, 503.1 [*M*−H, ^10^B, ^35^Cl, ^37^Cl, ^37^Cl, 12 %]^−^, 504.1 [*M*−H, ^11^B, ^35^Cl, ^37^Cl, ^37^Cl, 22 %]^−^; HRMS(ES^−^) (*m*/*z*): [*M*−H]^−^ calcd for C_19_H_12_^11^B^35^Cl_3_^19^F_6_NO, 499.9987; found, 499.9990, error: 0.6 ppm.

**2,2‐Bis(4‐methylphenyl)‐6‐methyl‐4‐(trichloromethyl)‐2,3‐dihydro‐1,3,2‐oxazaborinin‐1‐ium‐2‐uide (NBC38)**: EtOAc/*n*‐hexane, 1:19. Reaction scale: B(OMe)_3_ (0.28 mL, 2.50 mmol), 4‐bromotoluene (0.62 mL, 5.00 mmol) and magnesium turnings (0.13 g, 5.50 mmol) gives bis(4‐methylphenyl)borinic acid (0.44 g, 42 %). Bis(4‐methylphenyl)borinic acid (0.44 g, 2.11 mmol) and (*Z*)‐4‐amino‐5,5,5‐trichloropent‐3‐en‐2‐one (0.64 g, 3.16 mmol) gives NBC38 as a yellow solid (68.8 mg, 4 % over two steps); mp: 105–107 °C; ^1^H NMR (400 MHz, CDCl_3_): *δ*=7.41 (br s, 1 H, NH), 7.28 (d, *J*=7.2 Hz, 4 H, B‐Ar(*o*)), 7.13 (d, *J*=7.2 Hz, 4 H, B‐Ar(*m*)), 5.77 (s, 1 H, Cl_3_C(NH)C=C*H*), 2.34 (s, 6 H, CH_3×_2), 2.23 ppm (s, 3 H, CH_3_CO); ^13^C NMR (100 MHz, CDCl_3_): *δ*=186.2 (CH_3_ *C*O), 136.3 (B‐Ar(*p*)), 131.9 (B‐Ar(*o*)), 128.4 (B‐Ar(*m*)), 91.5 (Cl_3_C(NH)*C*=*C*H), 24.9 (CH_3_CO), 21.5 ppm (CH_3_), B‐Ar(*i*) and Cl_3_ *C*(NH)C=CH quaternary signals not observed; ^11^B{^1^H} NMR (128 MHz, CDCl_3_): *δ*=4.61 ppm; MS(ES^−^) (*m*/*z*): 392.1 [*M*−H, ^11^B, ^35^Cl, ^35^Cl, ^35^Cl, 100 %]^−^, 393.1 [*M*−H, ^10^B, ^35^Cl, ^35^Cl, ^37^Cl, 20 %]^−^, 394.1 [*M*−H, ^11^B, ^35^Cl, ^35^Cl, ^37^Cl, 88 %]^−^, 395.1 [*M*−H, ^10^B, ^35^Cl, ^37^Cl, ^37^Cl, 21 %]^−^, 396.1 [*M*−H, ^11^B, ^35^Cl, ^37^Cl, ^37^Cl, 22 %]^−^; MS(ES^+^) (*m*/*z*): 301.2 [*M*−tolyl, ^10^B, ^35^Cl, ^35^Cl, ^35^Cl, 75 %]^+^, 302.1 [*M*−tolyl, ^11^B, ^35^Cl, ^35^Cl, ^35^Cl, 90 %]^+^, 303.1 [*M*−tolyl, ^10^B, ^35^Cl, ^35^Cl, ^37^Cl, 28 %]^+^, 304.1 [*M*−tolyl, ^11^B, ^35^Cl, ^35^Cl, ^37^Cl, 100 %]^+^, 305.1 [*M*−tolyl, ^10^B, ^35^Cl, ^37^Cl, ^37^Cl, 16 %]^+^, 306.0 [*M*−tolyl, ^11^B, ^35^Cl, ^37^Cl, ^37^Cl, 25 %]^+^; HRMS(ES^−^) (*m*/*z*): [*M*−H]^−^ calcd for C_19_H_18_^11^B^35^Cl_3_NO, 392.0553; found, 392.0555, error: 0.6 ppm.

**2,2‐Bis(3,4‐dichlorophenyl)‐6‐methyl‐4‐(trichloromethyl)‐2,3‐dihydro‐1,3,2‐oxazaborinin‐1‐ium‐2‐uide (NBC39)**: EtOAc/*n*‐hexane, 1:19. Reaction scale: B(OMe)_3_ (0.28 mL, 2.50 mmol), 4‐bromo‐1,2‐dichlorobenzene (0.64 mL, 5.00 mmol) and magnesium turnings (0.13 g, 5.50 mmol) gives bis(3,4‐dichlorophenyl)borinic acid (0.92 g, 58 %). Bis(3,4‐dichlorophenyl)borinic acid (0.92 g, 2.88 mmol) and (*Z*)‐4‐amino‐5,5,5‐trichloropent‐3‐en‐2‐one (0.87 g, 4.32 mmol) gives NBC39 as a cream solid (0.71 g, 43 % over two steps); mp: 109–110 °C; ^1^H NMR (400 MHz, CDCl_3_): *δ*=7.37 (s, 2 H, B‐Ar‐H2), 7.36 (d, *J*=7.2 Hz, 2 H, B‐Ar‐H5), 7.10 (d, *J*=8.0 Hz, 2 H, B‐Ar‐H6), 5.88 (s, 1 H, Cl_3_C(NH)C=C*H*), 2.28 ppm (s, 3 H, CH_3_CO), NH signal is observed but overlapping with CHCl_3_ at 7.26 ppm; ^13^C NMR (100 MHz, CDCl_3_): *δ*=187.1 (CH_3_ *C*O), 166.7 (Cl_3_C(NH)*C*=*C*H), 133.5 (B‐Ar‐C6), 132.1 (B‐Ar‐C3), 131.1 (B‐Ar‐C4), 130.9 (B‐Ar‐C2), 129.9 (B‐Ar‐C5), 92.8 (Cl_3_C(NH)C=CH), 92.5 (CCl_3_), 24.9 ppm (*C*H_3_CO), B‐Ar‐C1 quaternary signal not observed; ^11^B{^1^H} NMR (128 MHz, CDCl_3_): *δ*=3.10 ppm; MS(ES^−^) (*m*/*z*): 499.9 [*M*−H, ^11^B, ^35^Cl, ^35^Cl, ^35^Cl, ^35^Cl, ^35^Cl, ^35^Cl, ^35^Cl, 45 %]^−^, 500.9 [*M*−H, ^10^B, ^35^Cl, ^35^Cl, ^35^Cl, ^35^Cl, ^35^Cl, ^35^Cl, ^37^Cl, 22 %]^−^, 501.9 [*M*−H, ^11^B, ^35^Cl, ^35^Cl, ^35^Cl, ^35^Cl, ^35^Cl, ^35^Cl, ^37^Cl, 100 %]^−^, 502.9 [*M*−H, ^10^B, ^35^Cl, ^35^Cl, ^35^Cl, ^35^Cl, ^35^Cl, ^37^Cl, ^37^Cl, 50 %]^−^, 503.9 [*M*−H, ^11^B, ^35^Cl, ^35^Cl, ^35^Cl, ^35^Cl, ^35^Cl, ^37^Cl, ^37^Cl, 98 %]^−^, 504.9 [*M*−H, ^10^B, ^35^Cl, ^35^Cl, ^35^Cl, ^35^Cl, ^37^Cl, ^37^Cl, ^37^Cl, 23 %]^−^, 505.9 [*M*−H, ^11^B, ^35^Cl, ^35^Cl, ^35^Cl, ^35^Cl, ^37^Cl, ^37^Cl, ^37^Cl, 63 %]^−^, 506.9 [*M*−H, ^10^B, ^35^Cl, ^35^Cl, ^35^Cl, ^37^Cl, ^37^Cl, ^37^Cl, ^37^Cl, 10 %]^−^, 507.9 [*M*−H, ^11^B, ^35^Cl, ^35^Cl, ^35^Cl, ^37^Cl, ^37^Cl, ^37^Cl, ^37^Cl, 21 %]^−^; HRMS(ES^−^) (*m*/*z*): [*M*−H]^−^ calcd for C_17_H_10_^11^B^35^Cl_7_NO, 499.8681; found, 499.8681, error: 0.0 ppm.

**5‐Acetyl‐6‐amino‐2,2‐bis(3‐chloro‐4‐methylphenyl)‐4‐(trichloromethyl)‐2,3‐dihydro‐1,3,2‐oxazaborinin‐1‐ium‐2‐uide (NBC40)**: Using an adapted procedure,^29^ *i*PrMgCl (2.0 m in THF, 1.21 mL, 2.42 mmol) was added dropwise to a solution of 2‐chloro‐4‐iodotoluene (0.28 mL, 1.98 mmol) in anhydrous THF (5 mL) in an oven‐dried Schlenk flask under N_2_. The reaction was stirred at 0 °C for 5 h. B(OMe)_3_ (0.10 mL, 0.92 mmol) was then added and the reaction mixture was stirred overnight allowing to warm to room temperature. HCl (3 m, 10 mL) was added and the reaction mixture was extracted with EtOAc (3×10 mL), washed with brine (1×10 mL), dried over MgSO_4_, filtered and evaporated in vacuo to give crude bis(3‐chloro‐4‐methylphenyl)borinic acid as a cream solid in quantitative yield. To a portion of this intermediate (0.20 g, 0.72 mmol) in anhydrous THF (5 mL) was added (*Z*)‐2‐acetyl‐3‐amino‐4,4,4‐trichlorobut‐2‐enamide (0.26 g, 1.08 mmol). The reaction was stirred at 50 °C for 16 h under N_2_. The reaction mixture was concentrated and purified by flash column chromatography (EtOAc/*n*‐hexane, 1:4). Collected fractions were evaporated in vacuo and stirred in the minimum amount of cold *n*‐hexane for 30 min to induce precipitation. The precipitate was then filtered and dried under vacuum to give NBC40 as a white solid (41.4 mg, 11 %); mp: 147–148 °C; ^1^H NMR (300 MHz, CDCl_3_): *δ*=11.22 (br s, 1 H, Cl_3_C(NH)C=C), 7.38 (s, 2 H, B‐Ar‐H2), 7.18 (d, *J*=7.8 Hz, 2 H, B‐Ar‐H6), 7.10 (d, *J*=7.2 Hz, 2 H, B‐Ar‐H5), 6.16 (br s, 1 H, CONH_2_), 5.89 (br s, 1 H, CONH_2_), 2.32 (s, 6 H, CH_3×_2), 2.26 ppm (s, 3 H, CH_3_CO); MS(ES^−^) (*m*/*z*): 502.1 [*M*−H, ^10^B, ^35^Cl, ^35^Cl, ^35^Cl, ^35^Cl, ^35^Cl, 10 %]^−^, 503.1 [*M*−H, ^11^B, ^35^Cl, ^35^Cl, ^35^Cl, ^35^Cl, ^35^Cl, 42 %]^−^, 504.1 [*M*−H, ^10^B, ^35^Cl, ^35^Cl, ^35^Cl, ^35^Cl, ^37^Cl, 27 %]^−^, 505.1 [*M*−H, ^11^B, ^35^Cl, ^35^Cl, ^35^Cl, ^35^Cl, ^37^Cl, 100 %]^−^, 506.1 [*M*−H, ^10^B, ^35^Cl, ^35^Cl, ^35^Cl, ^37^Cl, ^37^Cl, 24 %]^−^, 507.1 [*M*−H, ^11^B, ^35^Cl, ^35^Cl, ^35^Cl, ^37^Cl, ^37^Cl, 45 %]^−^, 508.1 [*M*−H, ^10^B, ^35^Cl, ^35^Cl, ^37^Cl, ^37^Cl, ^37^Cl, 14 %]^−^, 509.1 [*M*−H, ^11^B, ^35^Cl, ^35^Cl, ^37^Cl, ^37^Cl, ^37^Cl, 13 %]^−^; HRMS(ES^−^) (*m*/*z*): [*M*−H]^−^ calcd for C_20_H_17_^11^B^35^Cl_5_N_2_O_2_, 502.9831; found, 502.9833, error: 0.4 ppm.

**3‐Hydroxypyridine‐2‐carbonyloxy‐bis(3‐chloro‐4‐methylphenyl) borane (AN0128)**: Prepared according to a previously published method.^29^ *i*PrMgCl (2.0 m in THF, 2.42 mL, 4.83 mmol) was added dropwise to a solution of 2‐chloro‐4‐iodotoluene (0.56 mL, 3.96 mmol) in anhydrous THF (5 mL) in an oven‐dried Schlenk flask under N_2_. The reaction was stirred at 0 °C for 5 h. B(OMe)_3_ (0.21 mL, 1.84 mmol) was then added and the reaction mixture was stirred overnight allowing to warm to room temperature. HCl (3 m, 10 mL) was then added and the reaction mixture was extracted with EtOAc (3×10 mL), washed with brine (1×10 mL), dried over MgSO_4_, filtered and evaporated in vacuo to give crude bis(3‐chloro‐4‐methylphenyl)borinic acid as a cream solid in quantitative yield. A portion of this intermediate (0.50 g, 1.79 mmol) was dissolved in EtOH (5 mL) and heated at reflux. 3‐Hydroxypicolinic acid (0.20 g, 1.43 mmol) was added in portions to the hot solution and after the last addition, the reaction mixture was stirred at reflux for 15 min. The reaction was then cooled, resulting in the precipitation of product from the solution. The reaction mixture was partially concentrated and the precipitate was re‐crystallised in EtOH to give AN0128 as a white solid (0.30 g, 53 %); mp: 166–167 °C (lit.: 165.0–166.5 °C)^29^; ^1^H NMR (300 MHz, [D_6_]DMSO): *δ*=12.17 (br s, 1 H, OH), 8.58 (t, *J*=3.2 Hz, 1 H, Py‐H12), 7.93 (d, *J*=3.0 Hz, 2 H, Py‐H10,11), 7.22 (d, *J*=7.5 Hz, 2 H, B‐Ar‐H6), 7.20 (s, 2 H, B‐Ar‐H2), 7.11 (d, *J*=7.5 Hz, 2 H, B‐Ar‐H5), 2.27 ppm (s, 6 H, CH_3_×2); ^13^C NMR (100 MHz, [D_6_]DMSO): *δ*=162.2 (COO), 156.2 (Py‐C9), 134.2 (Py‐C8), 134.0 (Py‐C12), 133.0 (B‐Ar‐C4), 132.1 (B‐Ar‐C3), 131.9 (B‐Ar‐C6), 131.5 (Py‐C11), 130.7 (B‐Ar‐C5), 130.5 (B‐Ar‐C2), 127.5 (Py‐C10), 19.4 ppm (CH_3_), B‐Ar‐C1 quaternary signal not observed; ^11^B{^1^H} NMR (128 MHz, [D_6_]DMSO): *δ*=6.76 ppm; MS(ES^−^) (*m*/*z*): 397.1 [*M*−H, ^10^B, ^35^Cl, ^35^Cl, 22 %]^−^, 398.1 [*M*−H, ^11^B, ^35^Cl, ^35^Cl, 100 %]^−^, 399.1 [*M*−H, ^10^B, ^35^Cl, ^37^Cl, 63 %]^−^, 400.1 [*M*−H, ^11^B, ^35^Cl, ^37^Cl, 67 %]^−^, 401.1 [*M*−H, ^10^B, ^37^Cl, ^37^Cl, 17 %]^−^; HRMS(ES^−^) (*m*/*z*): [*M*−H]^−^ calcd for C_20_H_15_^11^B^35^Cl_2_NO_3_, 398.0528; found, 398.0526, error: 0.5 ppm. All data were in agreement with literature values.^29^

**3‐(1‐Amino‐2,2,2‐trifluoroethylidene)pentane‐2,4‐dione (8)**: Using a previously reported procedure,^33^ CF_3_CN was slowly generated by dropwise addition of a solution of trifluoroacetic anhydride (8.33 mL, 59.93 mmol) in anhydrous pyridine (40 mL) to a solution of trifluoroacetamide (6.77 g, 59.93 mmol) in anhydrous pyridine (20 mL) under N_2_ in a three‐neck round‐bottom flask equipped with a N_2_ gas inlet and gas outlet. The gas outlet was connected to a two‐neck round‐bottom flask containing a solution of acetylacetone (0.51 mL, 4.99 mmol) and Zn(acac)_2_ (14.1 mg, 0.050 mmol) in anhydrous CH_2_Cl_2_ (10 mL) that was equipped with a dry ice condenser connected to a bubbler outlet. CF_3_CN was bubbled into the stirring solution at room temperature for several hours until complete consumption of the trifluoroacetic anhydride solution. The reaction was further stirred at room temperature for 16 h. The reaction mixture was extracted with CH_2_Cl_2_ (3×10 mL), washed with brine (1×10 mL), dried over MgSO_4_, filtered and evaporated in vacuo to give **8** as a white solid (0.86 g, 88 %). ^1^H NMR (400 MHz, CDCl_3_): *δ*=2.46 (s, 3 H, CH_3_CO), 2.18 ppm (s, 3 H, CH_3_CO); ^13^C NMR (100 MHz, CDCl_3_): *δ*=202.1 (CH_3_ *C*O), 196.4 (CH_3_ *C*O), 145.7 (q, ^2^ *J*_CF_=33 Hz, F_3_C(NH_2_)*C*=*C*), 120.2 (q, ^1^ *J*_CF_=276 Hz, CF_3_), 112.8 (F_3_C(NH_2_)C=C), 32.2 (q, ^5^ *J*_CF_=4 Hz, *C*H_3_CO *cis* to CF_3_), 29.2 ppm (*C*H_3_CO); ^19^F{^1^H} NMR (376 MHz, CDCl_3_): *δ*=−66.4 ppm. All data were in agreement with literature values.^34^

**(*Z*)‐4‐Amino‐5,5,5‐trifluoropent‐3‐en‐2‐one (10)**: Using an adapted procedure,^34^ **8** (2.54 g, 13.02 mmol) was dissolved in EtOH (10 mL) and a saturated solution of K_2_CO_3_ (20 mL) was added. The reaction was stirred at 50 °C for 24 h. The reaction mixture was then extracted with CHCl_3_ (3×10 mL), washed with brine (1×10 mL), dried over MgSO_4_, filtered and evaporated in vacuo. The crude mixture was purified by flash column chromatography (1:4, EtOAc/*n*‐hexane) to give **10** as an orange solid (0.42 g, 27 % over two steps). ^1^H NMR (400 MHz, CDCl_3_): *δ*=5.52 (s, 1 H, F_3_C(NH_2_)C=C*H*), 2.18 ppm (s, 3 H, CH_3_CO); ^13^C NMR (100 MHz, CDCl_3_): *δ*=199.6 (CH_3_ *C*O), 147.1 (q, ^2^ *J*_CF_=33.3 Hz, F_3_C(NH_2_)*C*=*C*H), 120.4 (q, ^1^ *J*_CF_=274 Hz, CF_3_), 94.1 (q, ^3^ *J*_CF_=3.7 Hz, F_3_C(NH_2_)C=CH), 30.5 ppm (*C*H_3_CO); ^19^F{^1^H} NMR (376 MHz, CDCl_3_): *δ*=−71.8 ppm. All data were in agreement with literature values.^34^

**6‐Methyl‐2,2‐diphenyl‐4‐(trifluoromethyl)‐2,3‐dihydro‐1,3,2‐oxazaborinin‐1‐ium‐2‐uide (NBC41)**: Using an adapted procedure,^37^ **10** (0.29 g, 1.92 mmol) was added to a solution of DPBA (0.43 g, 1.23 mmol) in anhydrous THF (5 mL). The reaction mixture was stirred at 50 °C under Ar for 16 h. The reaction mixture was then concentrated and purified by flash column chromatography (3:20, EtOAc/*n*‐hexane) to give NBC41 as a yellow solid (0.34 g, 88 %); mp: 96–97 °C (lit.: 99–100 °C);^37^ ^1^H NMR (400 MHz, CDCl_3_): *δ*=7.14–7.31 (m, 10 H, B‐Ph×2), 6.95 (br s, 1 H, NH), 5.44 (d, ^4^ *J*_NH,H_=2.0 Hz, 1 H, F_3_C(NH)C=C*H*), 2.17 ppm (s, 3 H, CH_3_CO); ^13^C NMR (100 MHz, CDCl_3_): *δ*=188.3 (CH_3_ *C*O), 156.7 (q, ^2^ *J*_CF_=35.0 Hz, F_3_C(NH)*C*=*C*H), 131.8 (B‐Ph(*o*)), 127.6 (B‐Ph(*m*)), 127.1 (B‐Ph(*p*)), 118.8 (q, ^1^ *J*_CF_=276.7 Hz, CF_3_), 91.6 (F_3_C(NH)C=CH), 24.9 ppm (*C*H_3_CO); ^11^B{^1^H} NMR (128 MHz, CDCl_3_): *δ*=4.37 ppm; ^19^F{^1^H} NMR (376 MHz, CDCl_3_): *δ*=−72.9 ppm; MS(ES^−^) (*m*/*z*): 316.11 [*M*−H, 98 %]^−^; MS(ES^+^) (*m*/*z*): 340.11 [*M*+Na, 100 %]^+^; HRMS(ES^−^) (*m*/*z*): [*M*+Na]^−^ calcd for C_17_H_15_^11^BF_3_NO, 340.1091; found, 340.1085, error: 1.8 ppm. All data were in agreement with literature values.^37^

**(*Z*)‐2‐Acetyl‐3‐amino‐4,4,4‐trifluorobut‐2‐enamide (9)**: CF_3_CN was slowly generated by dropwise addition of a solution of trifluoroacetic anhydride (8.26 mL, 59.41 mmol) in anhydrous pyridine (40 mL) to a solution of trifluoroacetamide (6.71 g, 59.41 mmol) in anhydrous pyridine (20 mL) under N_2_ in a three‐neck round‐bottom flask equipped with a N_2_ gas inlet and gas outlet. The gas outlet was connected to a two‐neck round‐bottom flask containing a solution of acetoacetamide (1.00 g, 9.90 mmol) and Zn(acac)_2_ (27.9 mg, 0.099 mmol) in anhydrous CH_2_Cl_2_ (10 mL) that was equipped with a dry ice condenser connected to a bubbler outlet. CF_3_CN was bubbled into the stirring solution at room temperature for several hours until complete consumption of the trifluoroacetic anhydride solution. The reaction was further stirred at room temperature for 16 h. The reaction mixture was extracted with EtOAc (5×50 mL), washed with brine (3×50 mL), dried over MgSO_4_, filtered and evaporated in vacuo. Et_2_O (10 mL) was added to induce precipitation and the precipitate was filtered, washed with additional Et_2_O and dried to give **9** as a white solid (1.52 g, 78 %); mp: 192–193 °C; ^1^H NMR (400 MHz, CDCl_3_): *δ*=9.06 (br s, 2 H, NH_2_), 7.75 (br s, 1 H, CONH_2_), 7.40 (br s, 1 H, CONH_2_), 2.18 ppm (s, 3 H, CH_3_CO); ^13^C NMR (100 MHz, CDCl_3_): *δ*=196.5 (CH_3_ *C*O), 168.3 (CONH_2_), 144.9 (q, ^2^ *J*_CF_=32.3 Hz, F_3_C(NH_2_)*C*=*C*), 120.3 (q, ^1^ *J*_CF_=277.7 Hz, CF_3_), 107.0 (F_3_C(NH_2_)C=C), 28.0 ppm (CH_3_CO); ^19^F{^1^H} NMR (376 MHz, CDCl_3_): *δ*=−65.4 ppm; MS(ES^−^) (*m*/*z*): 195.04 [*M*−H, 100 %]^−^; MS(ES^+^) (*m*/*z*): 219.03 [*M*+Na, 100 %]^+^; HRMS(ES^−^) (*m*/*z*): [*M*+Na]^−^ calcd for C_6_H_7_F_3_N_2_O_2_, 219.0352; found, 219.0346, error: 2.7 ppm.

**5‐Acetyl‐6‐amino‐2,2‐diphenyl‐4‐(trifluoromethyl)‐2,3‐dihydro‐1,3,2‐oxazaborinin‐1‐ium‐2‐uide (NBC42)**: **9** (0.72 g, 3.69 mmol) was added to a solution of DPBA (0.43 g, 1.23 mmol) in anhydrous THF (5 mL). The reaction mixture was stirred at 50 °C under Ar for 16 h. The reaction mixture was then concentrated and purified by flash column chromatography (1:5, EtOAc/*n*‐hexane). The collected fractions were combined, evaporated in vacuo and precipitated in *n*‐hexane (10 mL) to give NBC42 as a white solid (0.28 g, 64 %); mp: 118–120 °C; ^1^H NMR (300 MHz, CDCl_3_): *δ*=10.05 (br s, 1 H, NH), 7.22–7.36 (m, 10 H, B‐Ph), 6.17 (br s, 1 H, NH), 2.26 ppm (q, ^6^ *J*_HF_=2.1 Hz, 3 H, CH_3_CO); ^13^C NMR (75 MHz, CDCl_3_): *δ*=196.3 (CH_3_ *C*O), 169.8 (CONH_2_), 156.7 (q, ^2^ *J*_CF_=34.6 Hz, F_3_C(NH)*C*=*C*), 131.8 (B‐Ar(*o*)), 127.5 (B‐Ar(*m*)), 127.0 (B‐Ar(*p*)), 119.4 (q, ^1^ *J*_CF_=280.0 Hz, CF_3_), 97.2 (F_3_C(NH)C=C), 30.5 ppm (q, ^5^ *J*_CF_=5.5 Hz, *C*H_3_CO), B‐Ar(*i*) quaternary signal not observed; ^11^B{^1^H} NMR (128 MHz, CDCl_3_): *δ*=2.55 ppm; ^19^F{^1^H} NMR (376 MHz, CDCl_3_): *δ*=−65.1 ppm; MS(ES^−^) (*m*/*z*): 227.0 [*M*‐(BPh_2_)‐H, 50 %]^−^, 359.1 [*M*−H, 100 %]^−^, 523.3 [*M*+BPh_2_‐H, 60 %]^−^; MS(ES^+^) (*m*/*z*): 219.0 [*M*‐BPh_2_+Na, 50 %]^+^, 399.1 [*M*+K, 60 %]^+^, 219.0 [*M*+BPh_2_+Na, 40 %]^+^, 563.2 [*M*+BPh_2_+K, 100 %]^+^; HRMS(ES^+^) (*m*/*z*): [*M*+H]^+^ calcd for C_18_H_17_^11^BF_3_N_2_O_2_, 361.1330; found, 361.1338, error: 2.3 ppm.

### Biology

**Cell culture**: Immortalised murine bone marrow‐derived macrophages (iBMDMs) were cultured in DMEM, 10 % fetal bovine serum (FBS), 100 U mL^−1^ penicillin and 100 μg mL^−1^ streptomycin (PenStrep). Cells were seeded overnight at 0.75×10^6^ cells per mL and then stimulated with LPS (*E. coli* O26:B6, 1 μg mL^−1^, 4 h), and then incubated with vehicle (0.5 % DMSO) or drug as indicated (10 μm) for 15 min before activation of NLRP3 using nigericin (10 μm, 60 min). IL‐1β release was measured by a specific ELISA (R&D systems).

**Data presentation and statistical analysis**: Data are presented as mean values ± standard error of the mean (SEM) of at least three separate experiments. Statistical analyses performed were one‐way analysis of variance (ANOVA) with Dunnett's multiple comparisons test post hoc. Accepted levels of significance were *p<0.05, ***p<0.001. Statistical analyses were carried out using GraphPad Prism.

## Conflict of interest

*The authors declare no conflict of interest*.

## Supporting information

As a service to our authors and readers, this journal provides supporting information supplied by the authors. Such materials are peer reviewed and may be re‐organized for online delivery, but are not copy‐edited or typeset. Technical support issues arising from supporting information (other than missing files) should be addressed to the authors.

Supplementary
