# Supplementary material for: Design, Synthesis and Evaluation of Oxazaborine Inhibitors of the NLRP3 Inflammasome
Source: ChemMedChem. 2018 Feb 5;13(4):312–20. doi: 10.1002/cmdc.201700731 (PMC5947144; doi:10.1002/cmdc.201700731)
Supplement: Supplementary file 1 — Supplementary [file CMDC-13-312-s001.pdf]

CHEMMEDCHEM Chemistry & Drug Discovery

Supporting Information

Design, Synthesis and Evaluation of Oxazaborine Inhibitors of the NLRP3 Inflammasome

Alex G. Baldwin,[a] Victor S. Tapia,[b] Tessa Swanton,[b] Claire S. White,[b] James A. Beswick,[a] David Brough,*[b] and Sally Freeman*[a]

cmdc_201700731_sm_miscellaneous_information.pdf

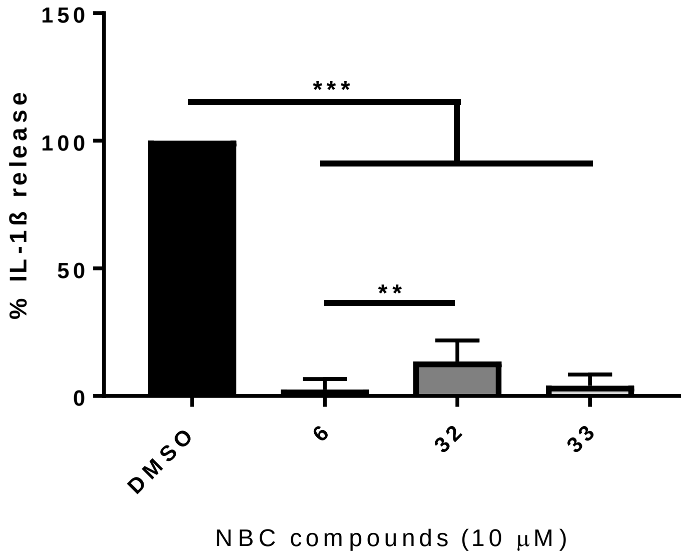

Figure S1: Effects of NBC32 and NBC33 on IL-1β release. THP-1 cells were primed with LPS (1 µg/ml, 4 h) and then treated with vehicle or NBC molecule (NBC6, NBC32 or NBC33) at 10 µM for 15 min before stimulation with nigericin (10 µM, 1h). IL-1β was measured by ELISA. The effects of the NBC molecules on IL-1β release was normalised to nigericin-induced IL-1β in the absence of any inhibitor. Data are presented as the mean ± s.e.m. with a minimum of 3 experiments per group. Statistical analysis was performed using a one-way analysis of variance (ANOVA) followed by a Tukey's multiple comparison test.

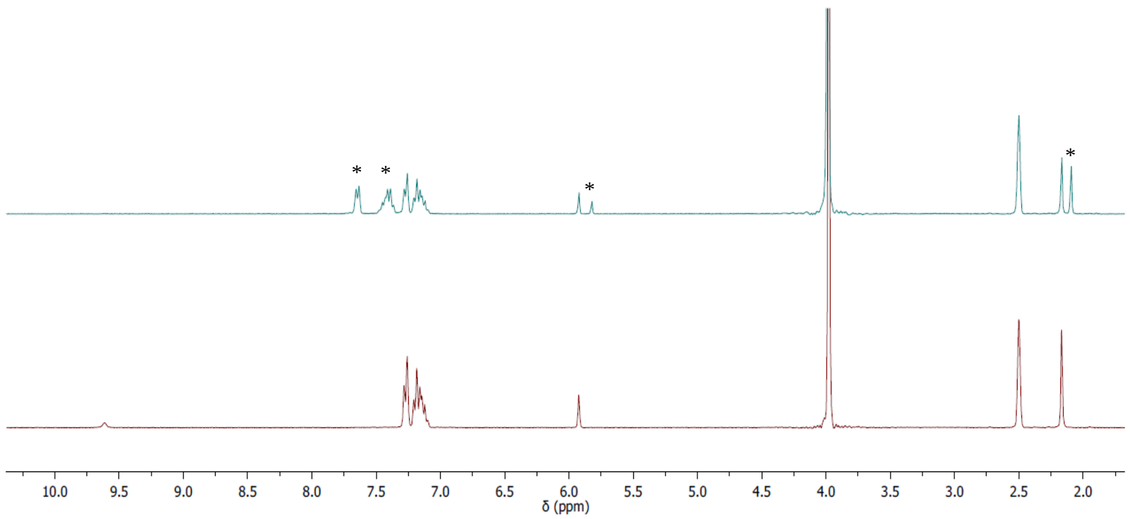

Figure S2: Stacked ^{1}H NMR spectra of BC23 (0.02 mmol) in 9:1 DMSO-d_6/D_2O (700 μL) at 37 ^{o}C; red: 0 h, blue: 24 h. Chemical shifts are defined in parts per million and referenced against residual undeuterated DMSO-d_6 (δ = 2.50 ppm). * Indicates new peaks associated with hydrolysis products of BC23.

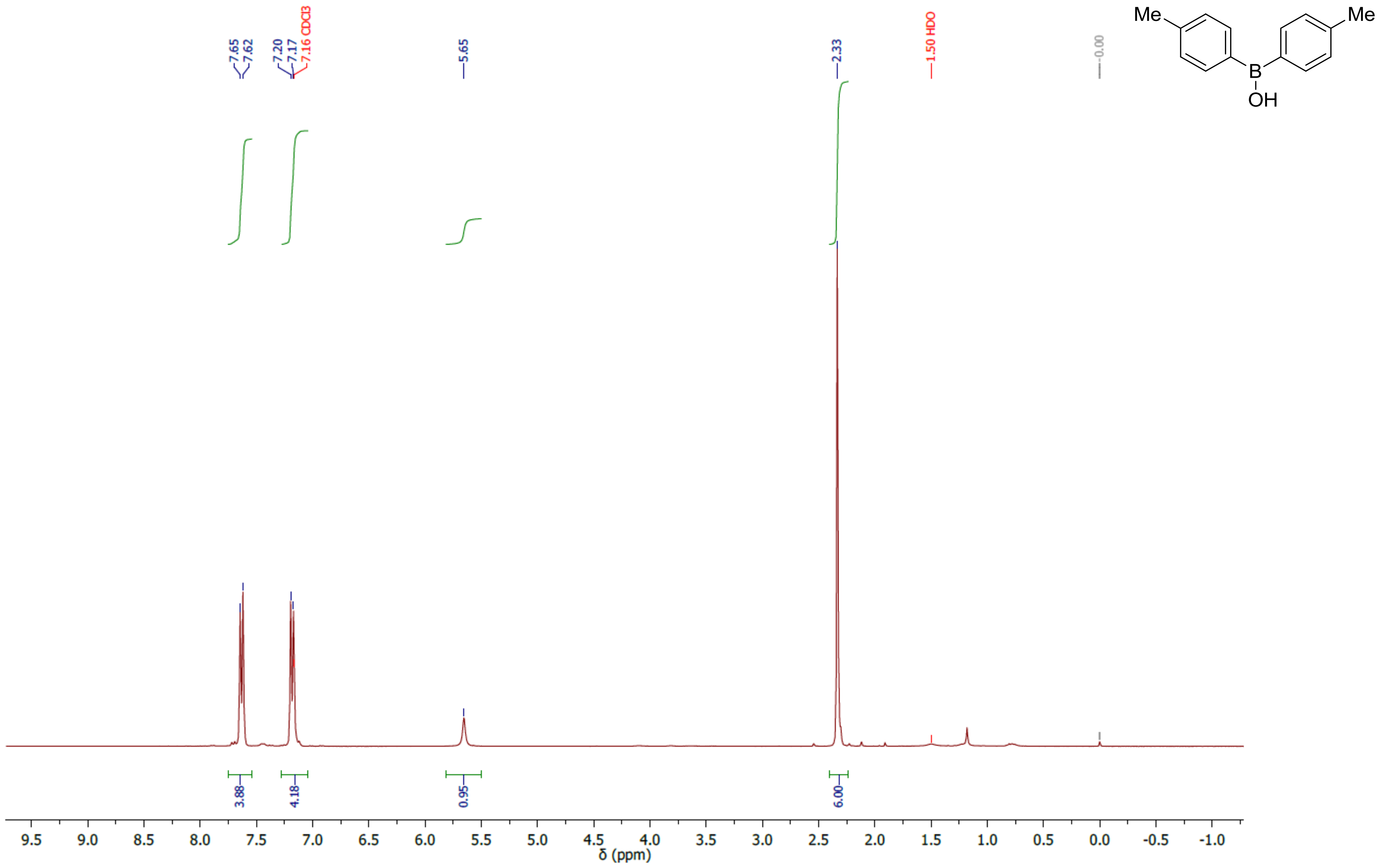

^{1}H NMR spectrum ($CDCl_3$) of di-*p*-tolylborinic acid (**5a**)

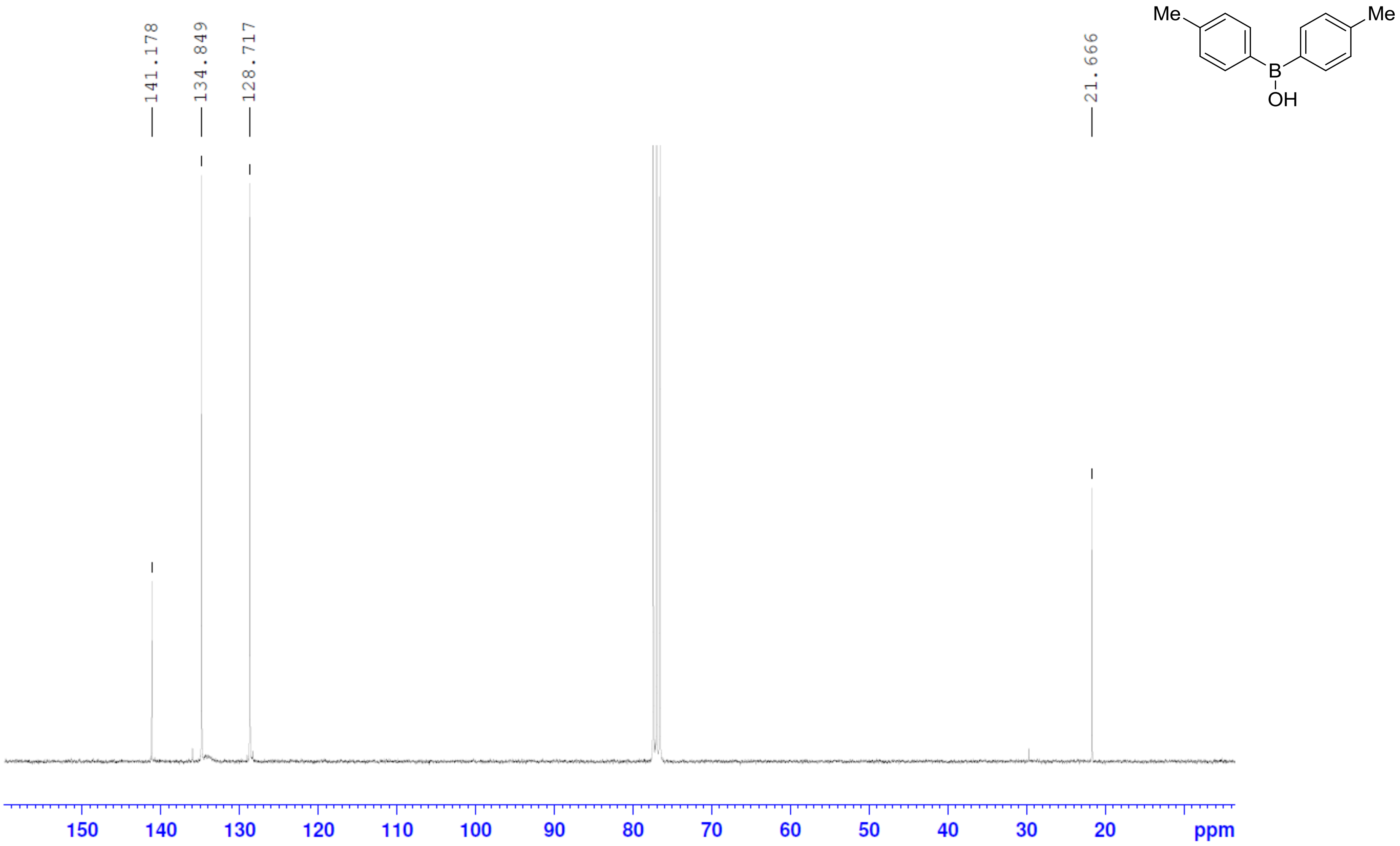

^{13}C NMR spectrum ($CDCl_3$) of di-*p*-tolylborinic acid (**5a**)

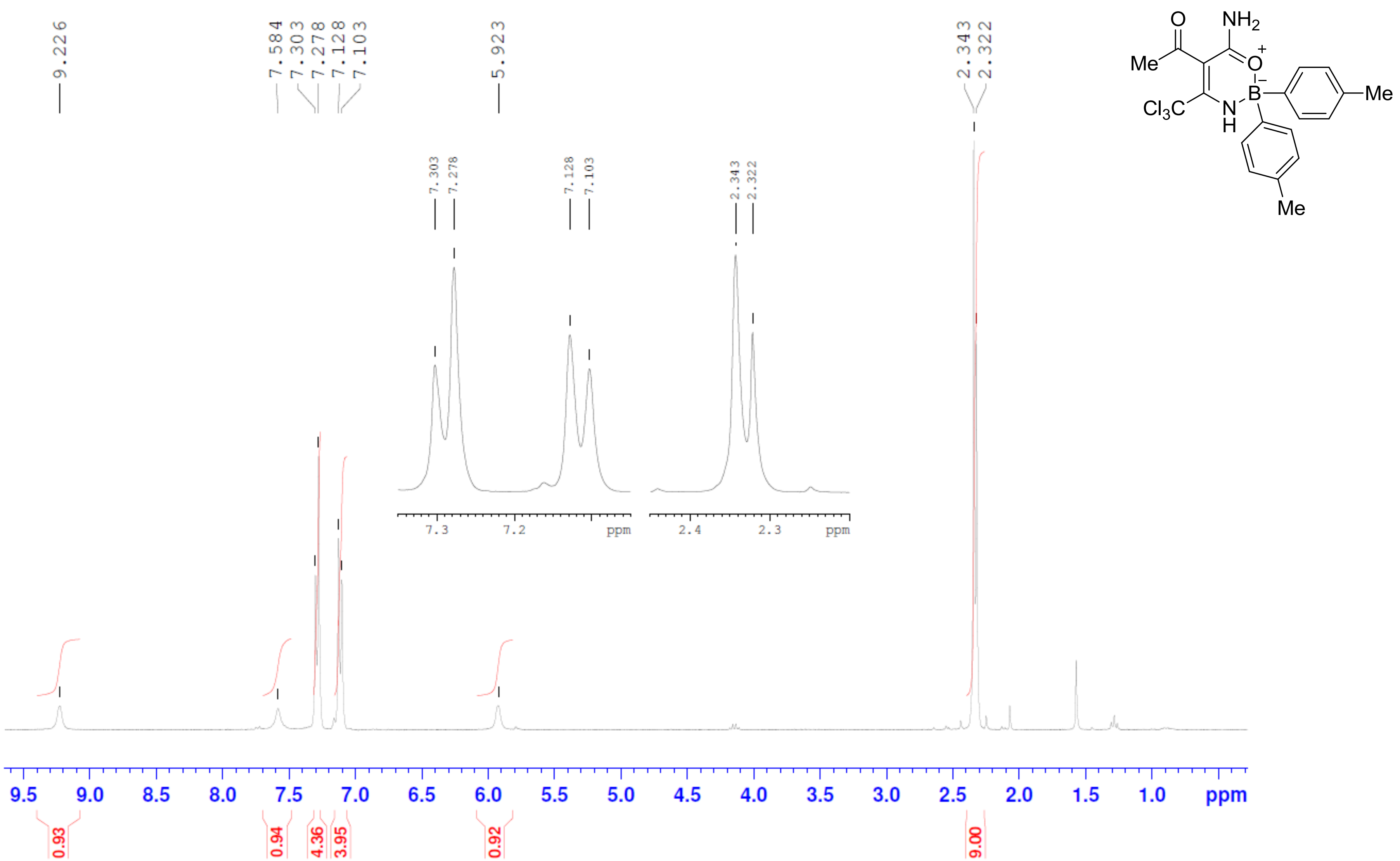

^{1}H NMR spectrum ($CDCl_3$) of 5-acetyl-6-amino-2,2-bis(*p*-tolyl)-4-(trichloromethyl)-2,3-dihydro-1,3,2-oxazaborinin-1-ium-2-uide (NBC32)

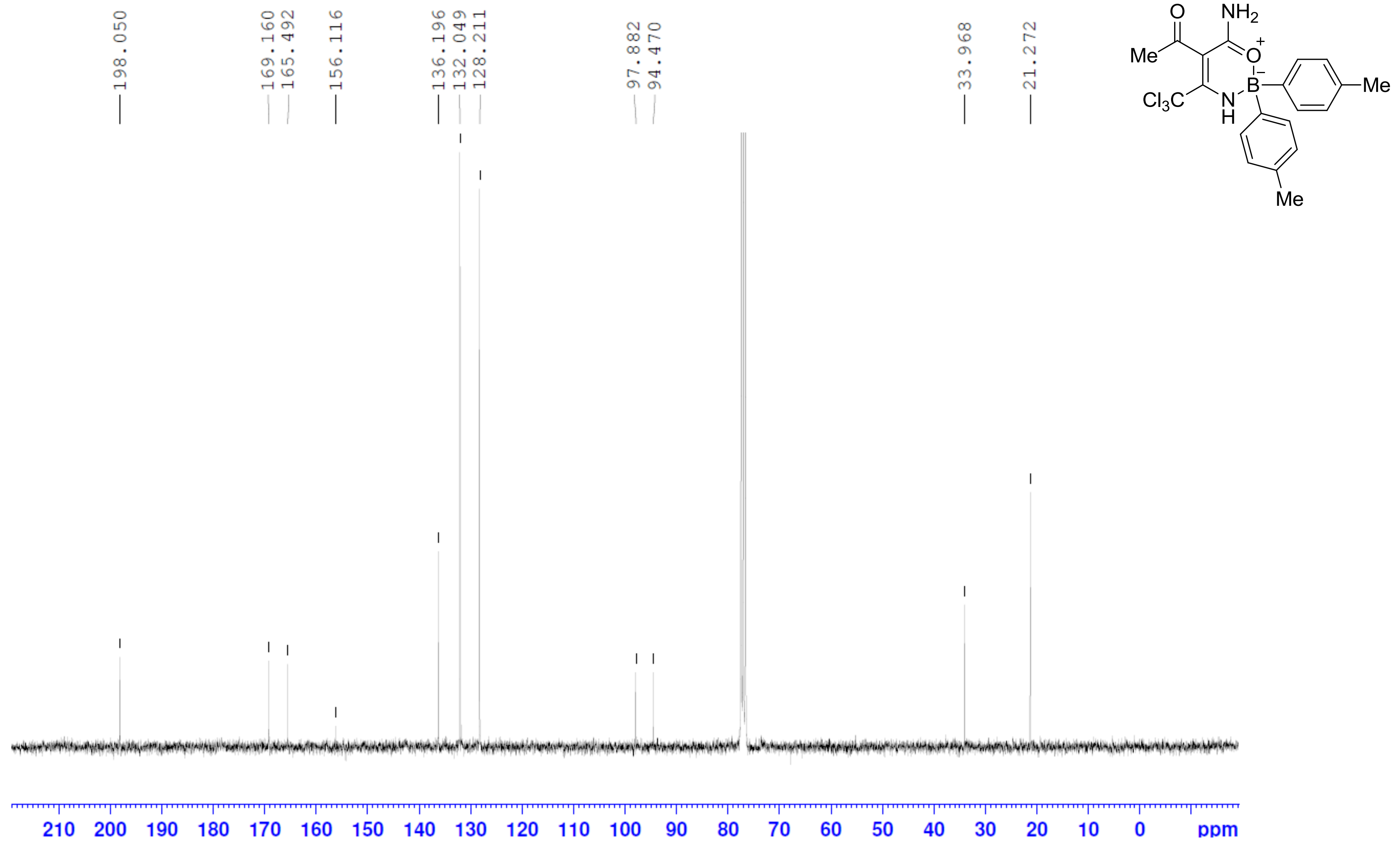

^{13}C NMR spectrum ($CDCl_3$) of 5-acetyl-6-amino-2,2-bis(*p*-tolyl)-4-(trichloromethyl)-2,3-dihydro-1,3,2-oxazaborinin-1-ium-2-uide (NBC32)

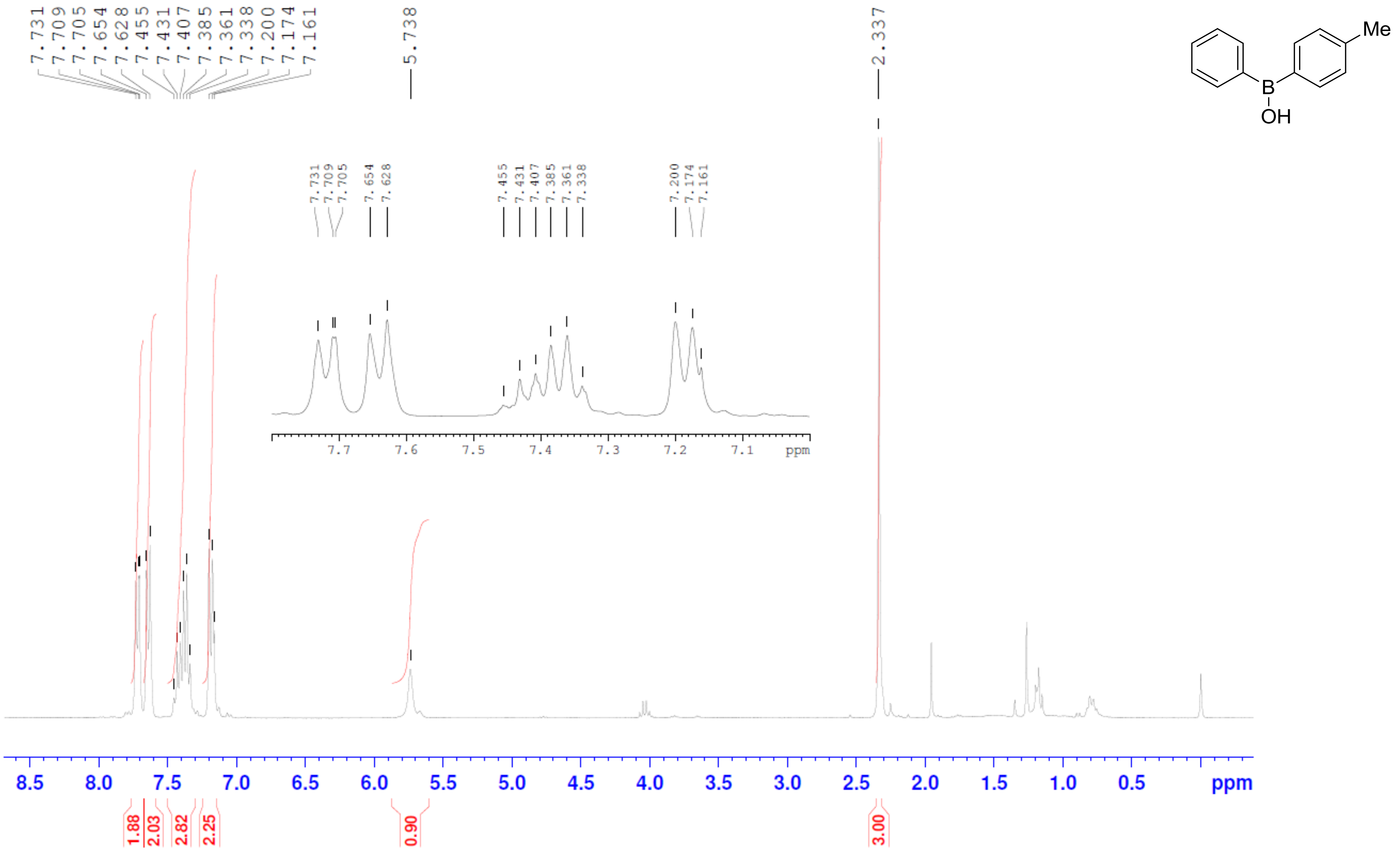

^{1}H NMR spectrum ($CDCl_3$) of (phenyl)(*p*-tolyl)borinic acid (**6a**)

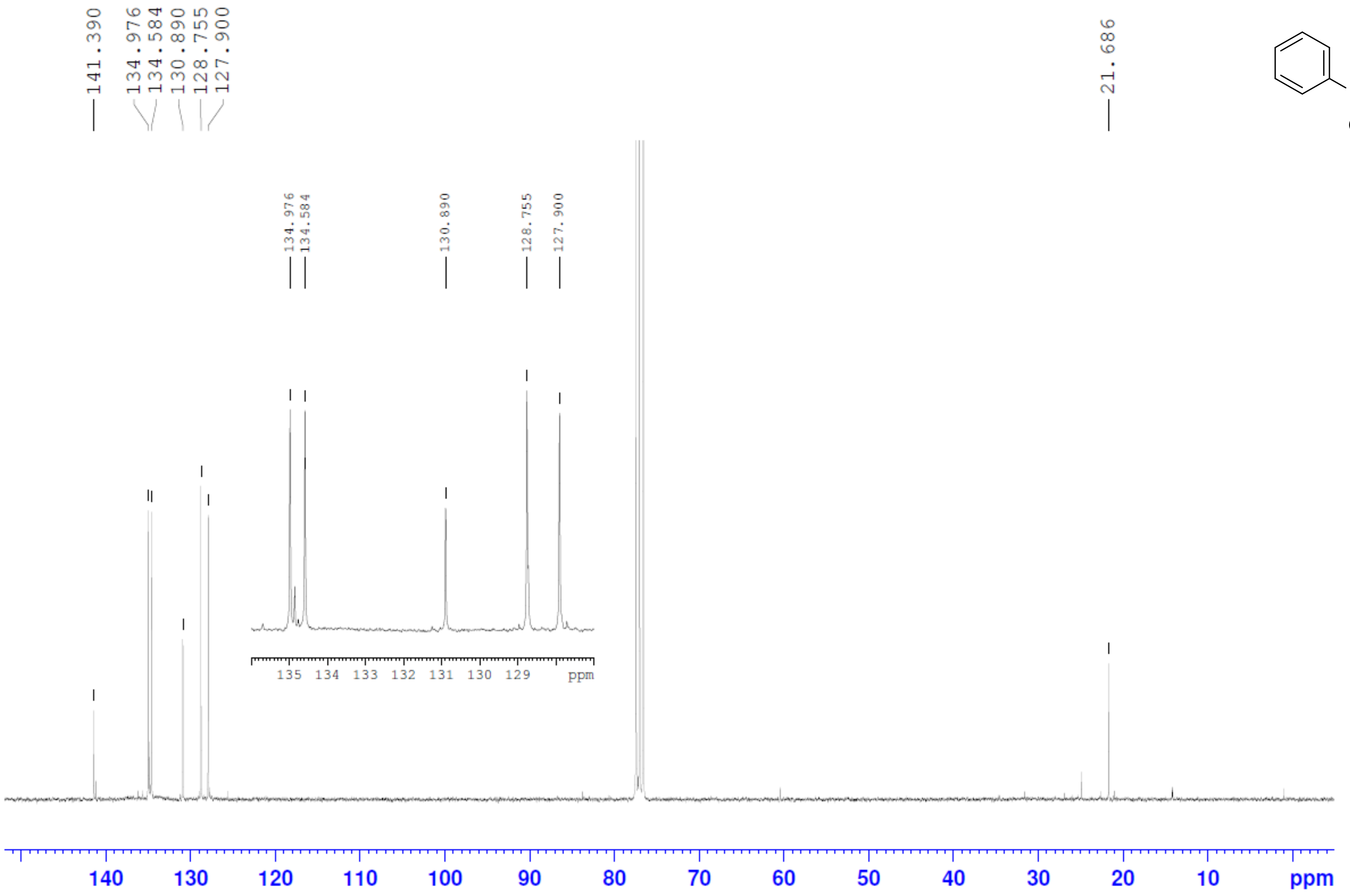

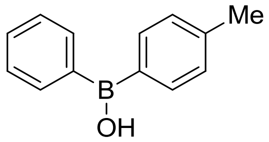

^{13}C NMR spectrum ($CDCl_3$) of (phenyl)(*p*-tolyl)borinic acid (**6a**)

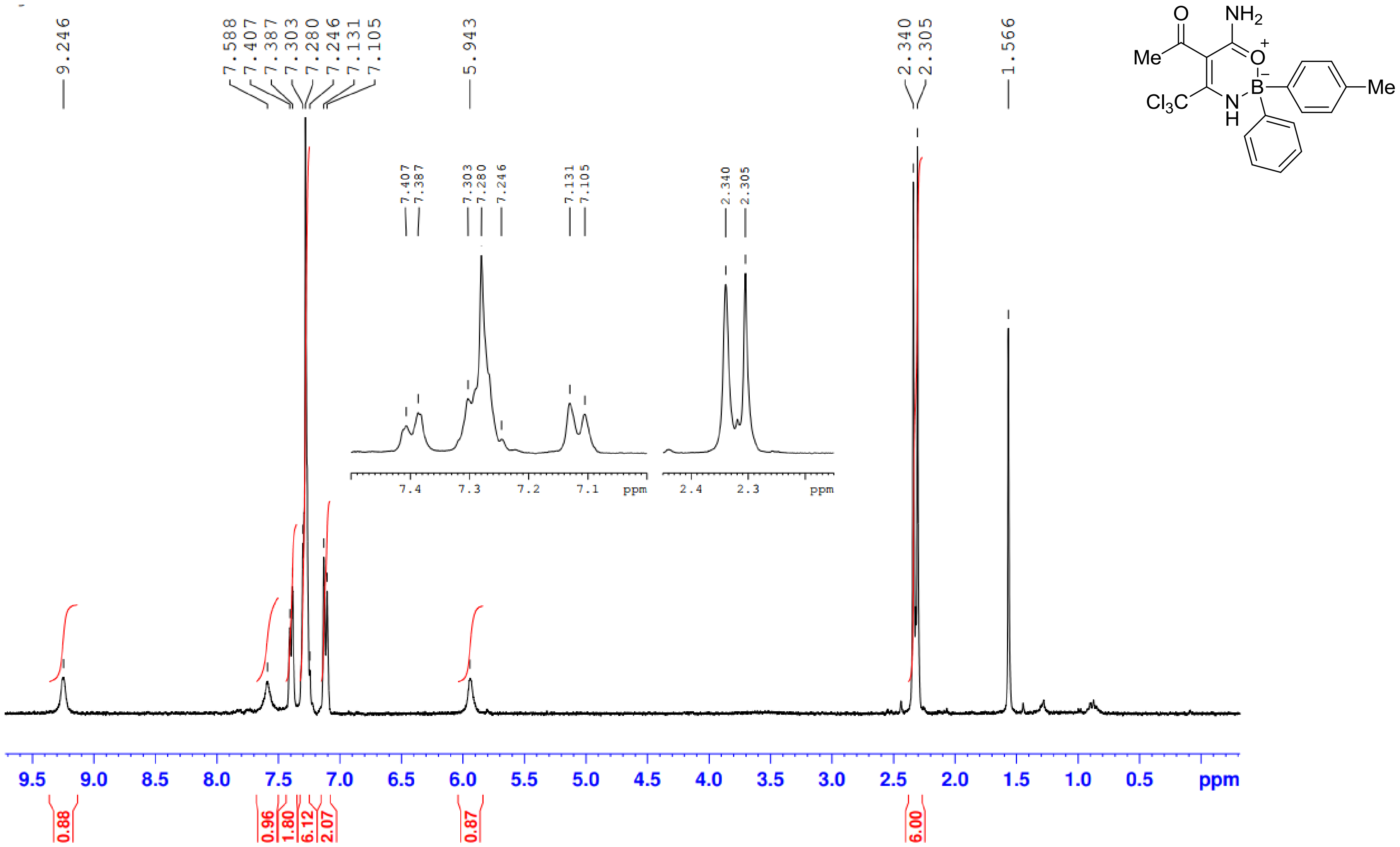

^{1}H NMR spectrum ($CDCl_3$) of 5-acetyl-6-amino-2-(phenyl)-2-(*p*-tolyl)-4-(trichloromethyl)-2,3-dihydro-1,3,2-oxazaborinin-1-ium-2-uide (NBC33)

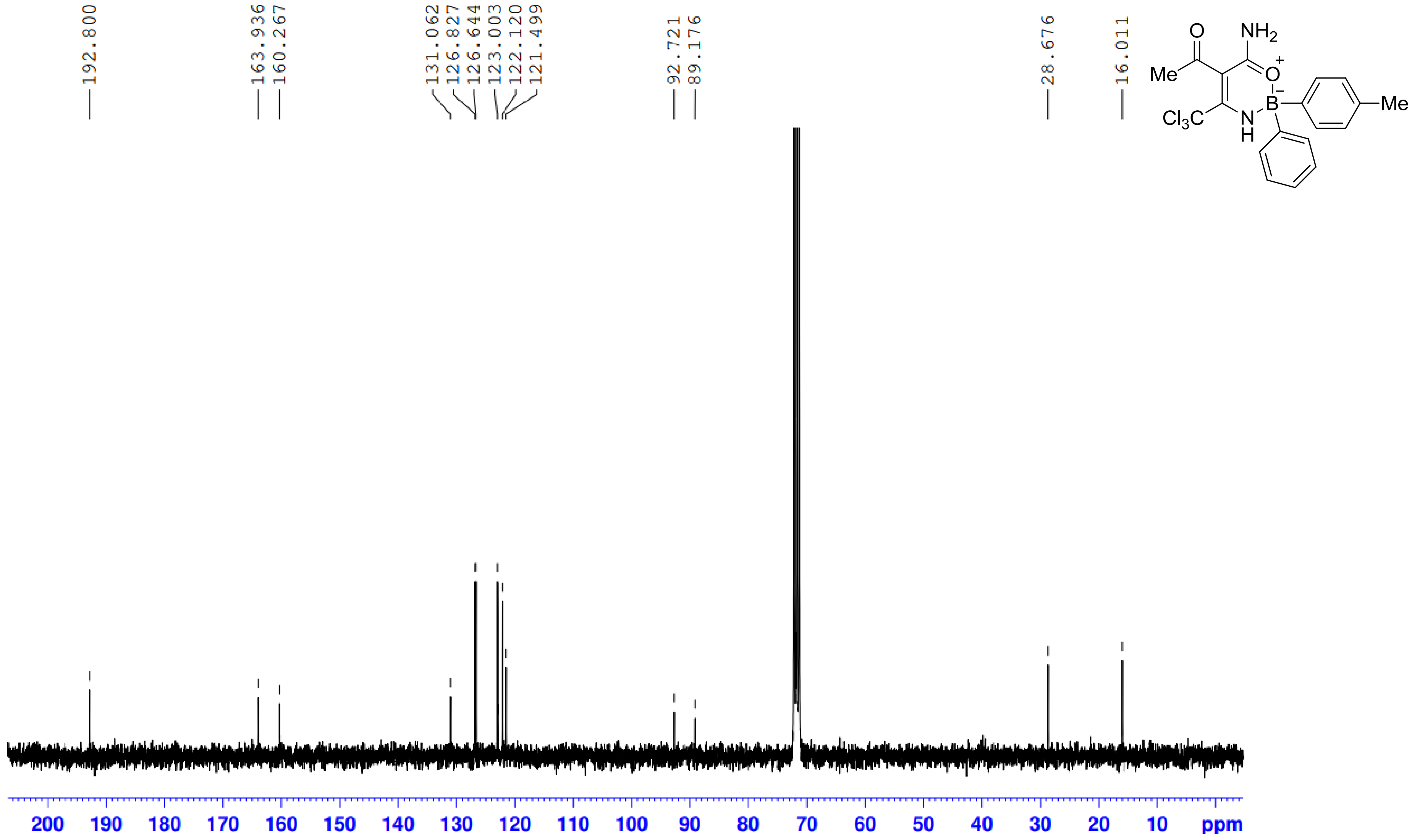

^{13}C NMR spectrum ($CDCl_3$) of 5-acetyl-6-amino-2-(phenyl)-2-(*p*-tolyl)-4-(trichloromethyl)-2,3-dihydro-1,3,2-oxazaborinin-1-ium-2-uide (NBC33)

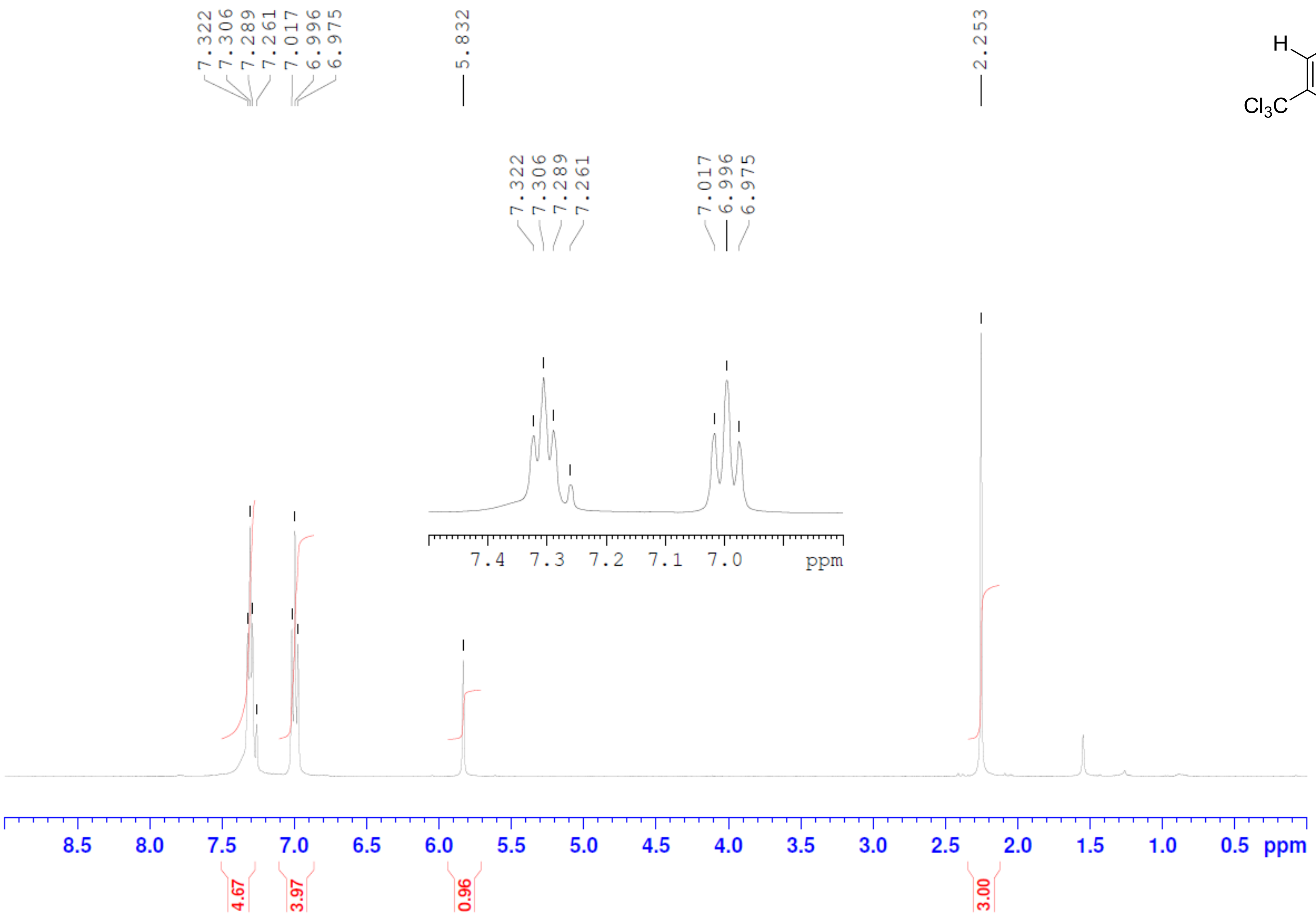

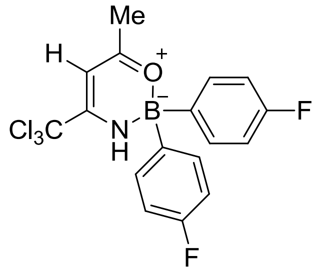

^{1}H NMR spectrum ($CDCl_3$) of 2,2-bis(4-fluorophenyl)-6-methyl-4-(trichloromethyl)-2,3-dihydro-1,3,2-oxazaborinin-1-ium-2-uide (NBC35)

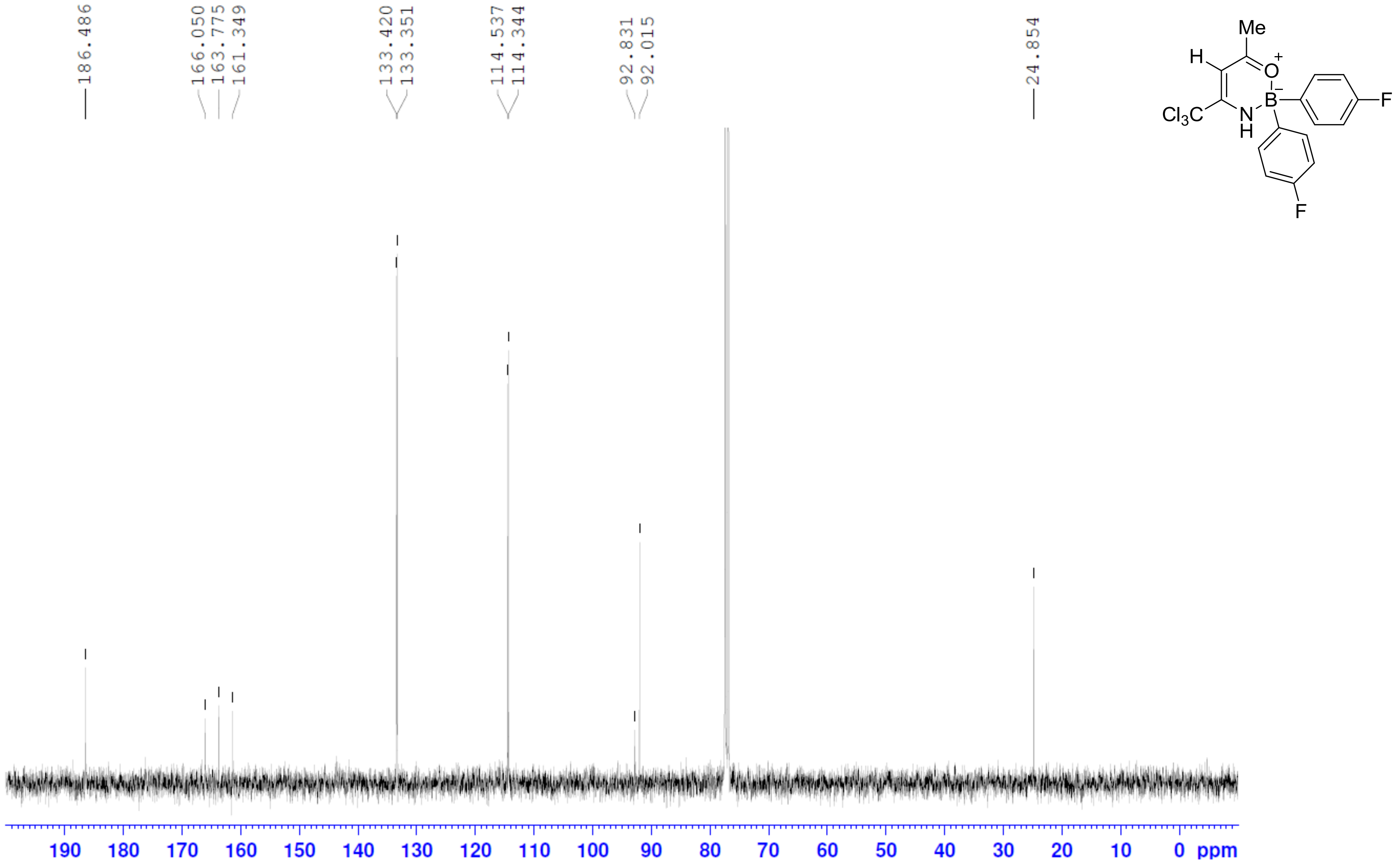

^{13}C NMR spectrum ($CDCl_3$) of 2,2-bis(4-fluorophenyl)-6-methyl-4-(trichloromethyl)-2,3-dihydro-1,3,2-oxazaborinin-1-ium-2-uide (NBC35)

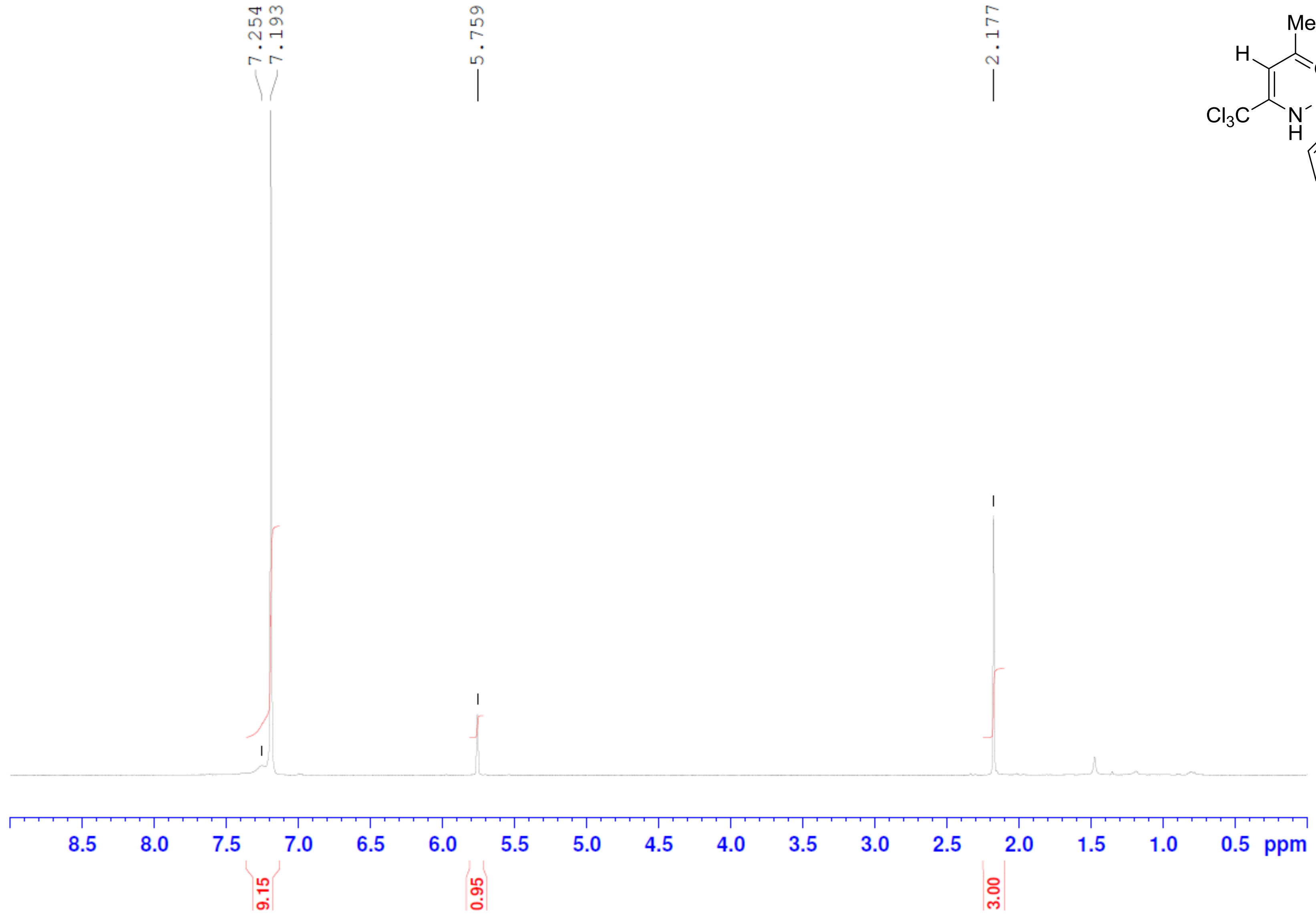

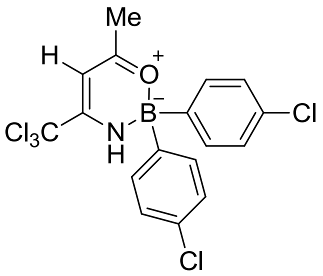

^{1}H NMR spectrum ($CDCl_3$) of 2,2-bis(4-chlorophenyl)-6-methyl-4-(trichloromethyl)-2,3-dihydro-1,3,2-oxazaborinin-1-ium-2-uide (NBC36)

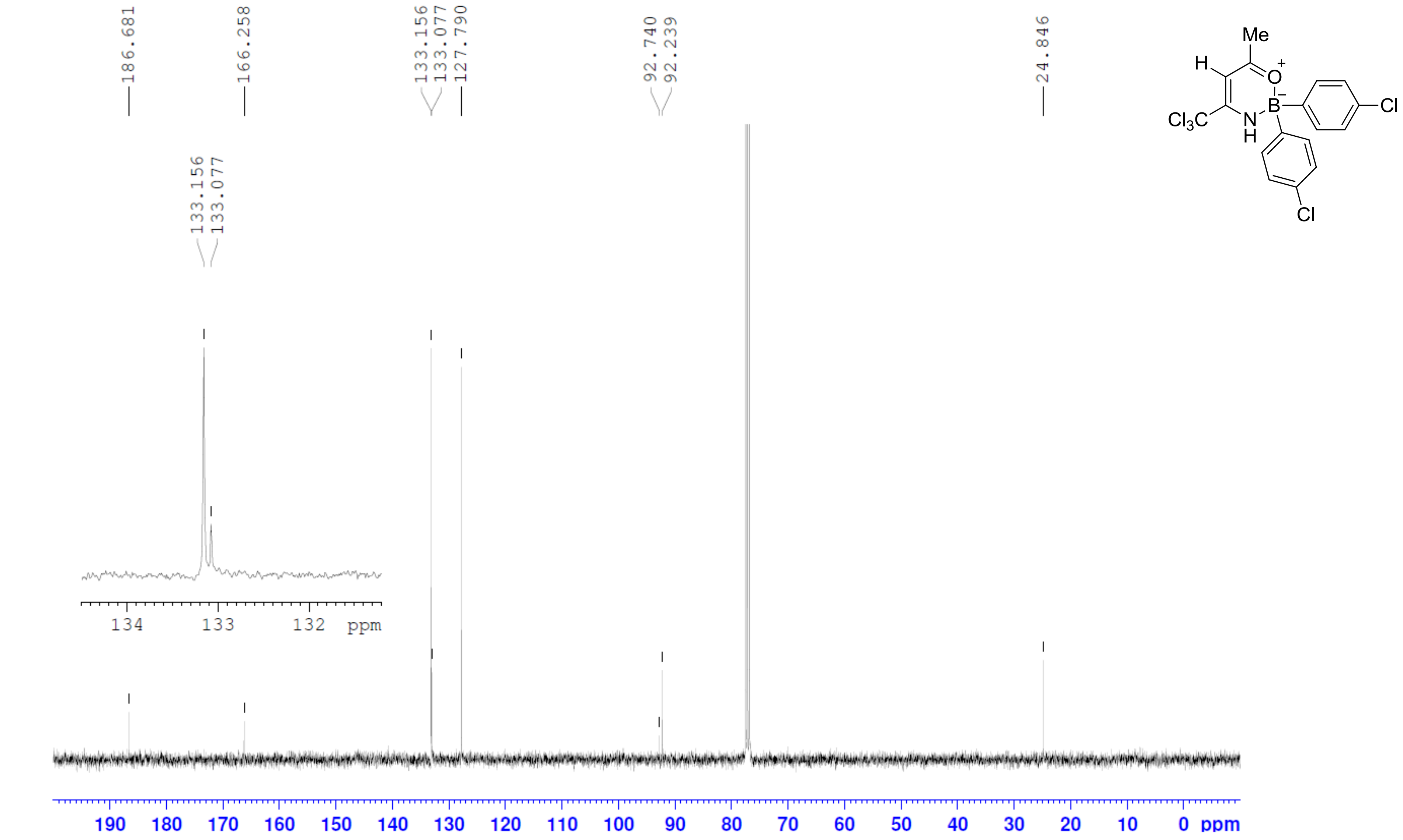

^{13}C NMR spectrum ($CDCl_3$) of 2,2-bis(4-chlorophenyl)-6-methyl-4-(trichloromethyl)-2,3-dihydro-1,3,2-oxazaborinin-1-ium-2-uide (NBC36)

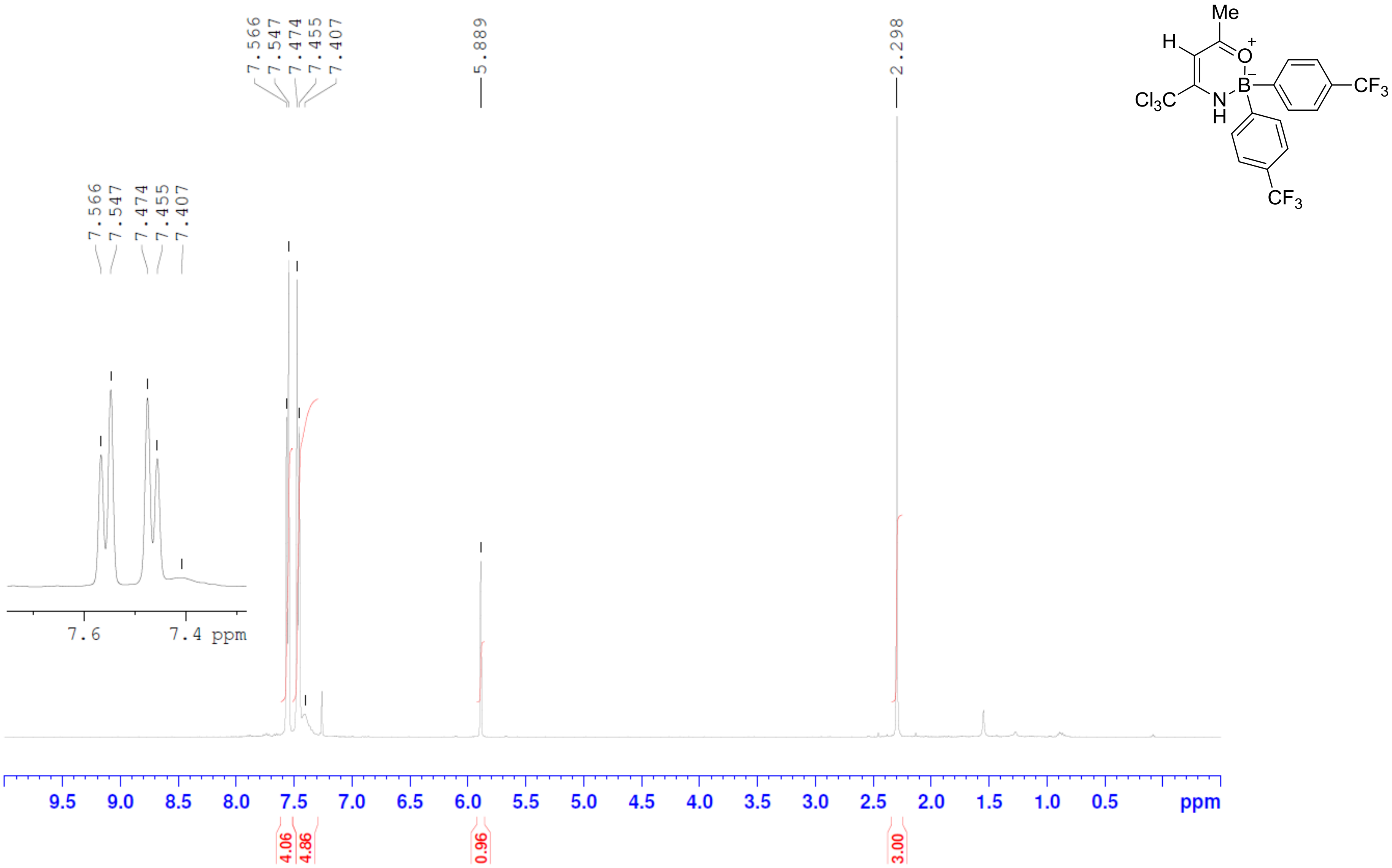

^{1}H NMR spectrum ($CDCl_3$) of 6-methyl-4-(trichloromethyl)-2,2-bis(4-(trifluoromethyl)phenyl)-2,3-dihydro-1,3,2-oxazaborinin-1-ium-2-uide (NBC37)

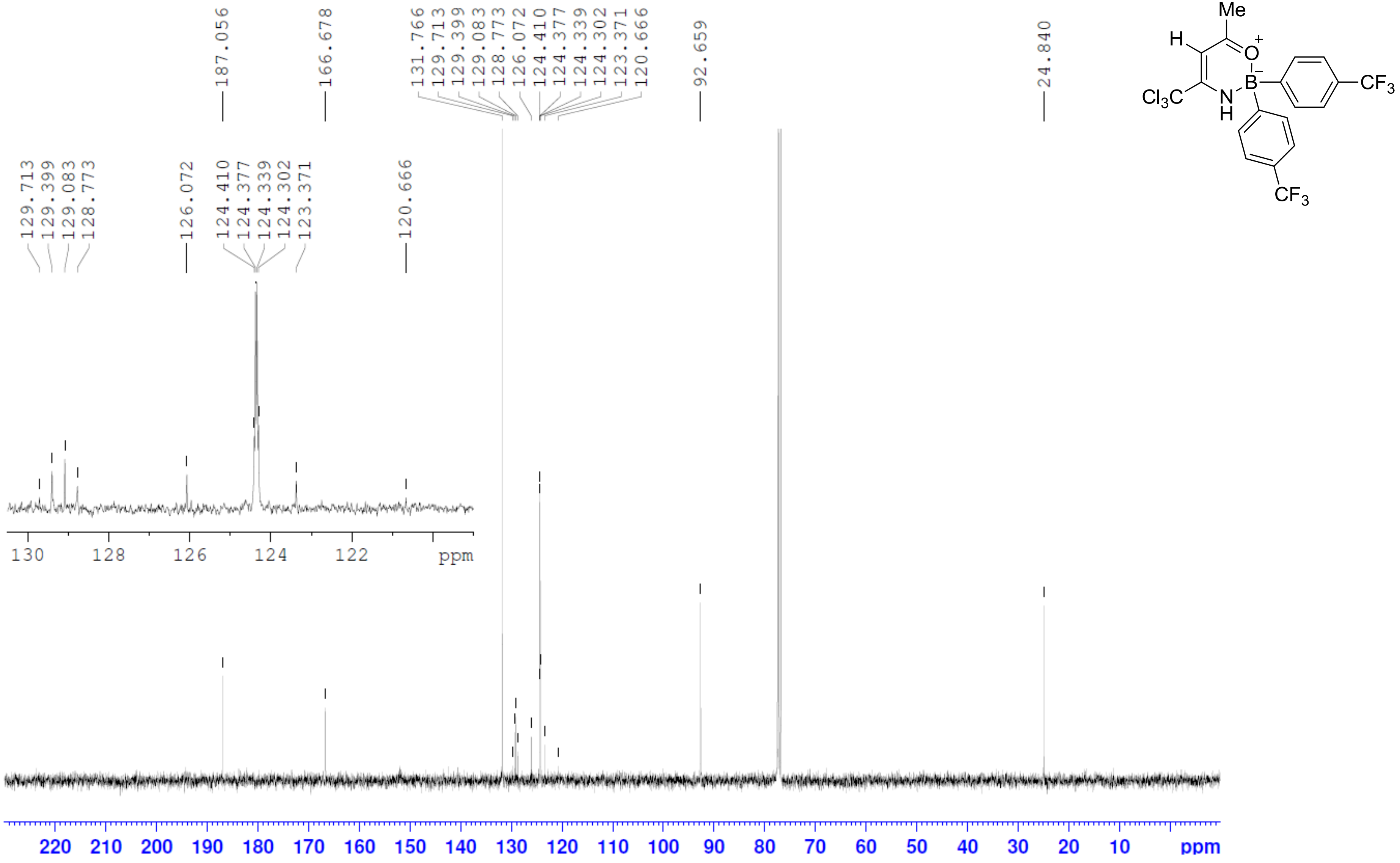

^{13}C NMR spectrum ($CDCl_3$) of 6-methyl-4-(trichloromethyl)-2,2-bis(4-(trifluoromethyl)phenyl)-2,3-dihydro-1,3,2-oxazaborinin-1-ium-2-uide (NBC37)

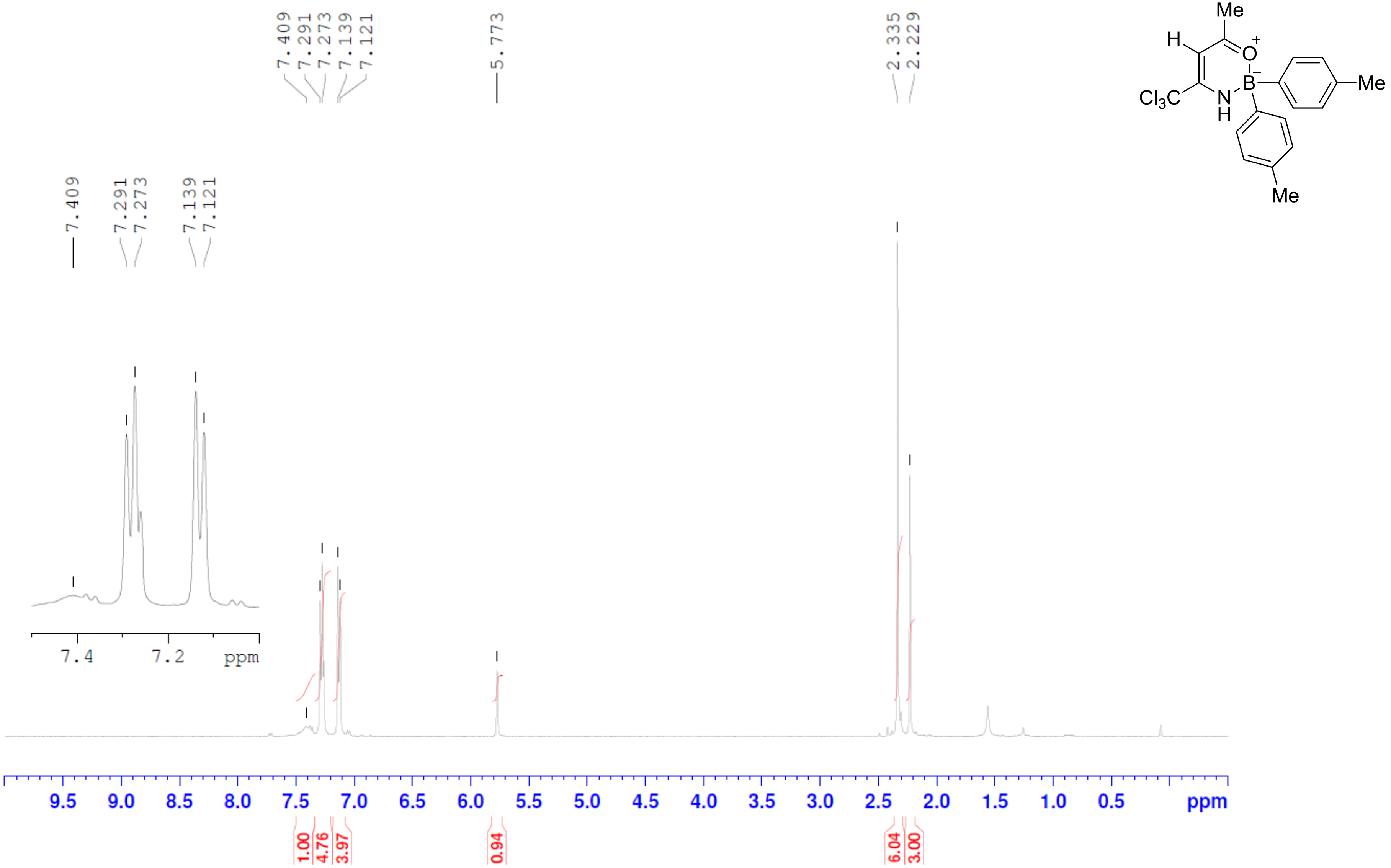

^{1}H NMR spectrum ($CDCl_3$) of 2,2-bis(4-methylphenyl)-6-methyl-4-(trichloromethyl)-2,3-dihydro-1,3,2-oxazaborinin-1-ium-2-uide (NBC38)

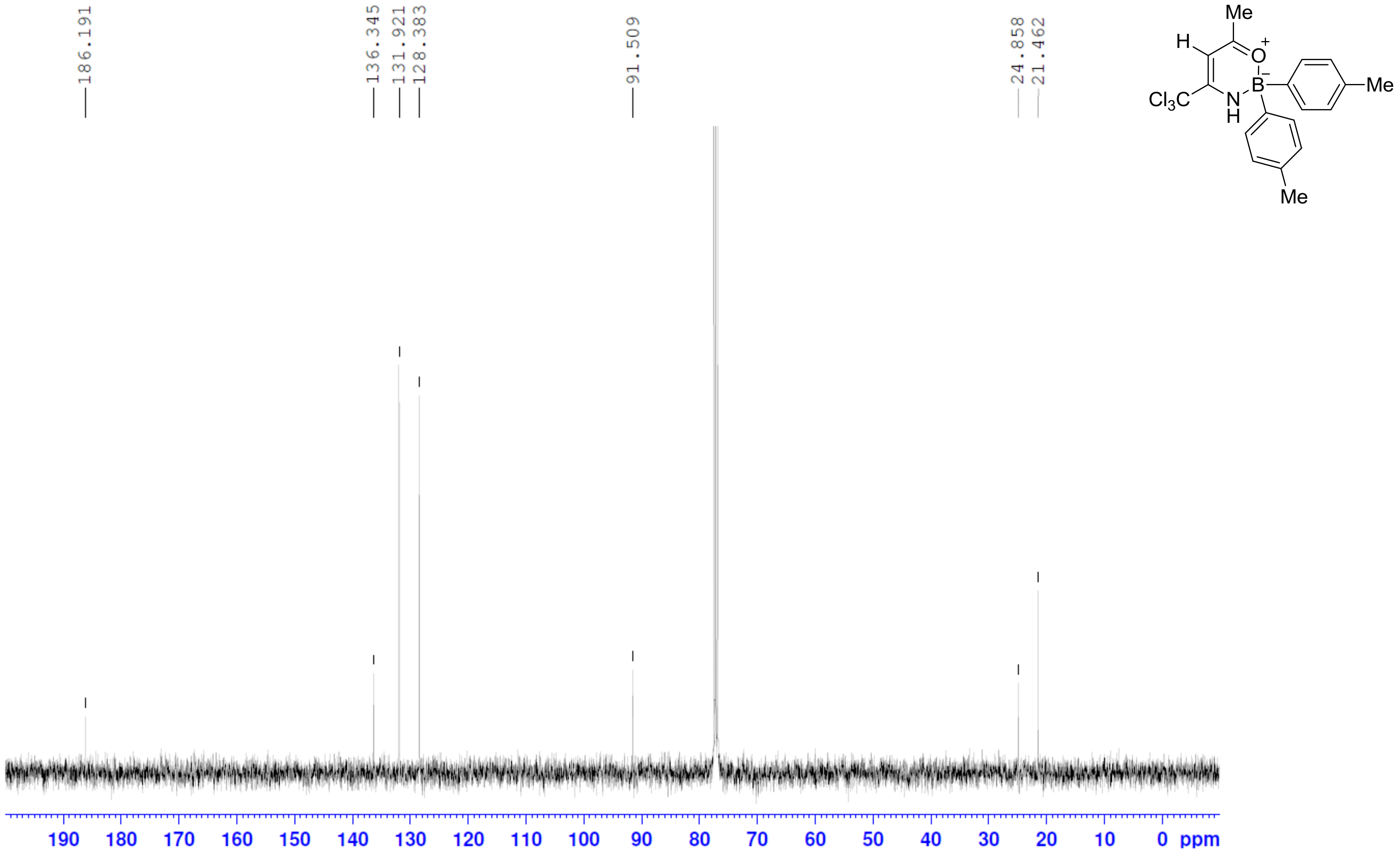

^{13}C NMR spectrum ($CDCl_3$) of 2,2-bis(4-methylphenyl)-6-methyl-4-(trichloromethyl)-2,3-dihydro-1,3,2-oxazaborinin-1-ium-2-uide (NBC38)

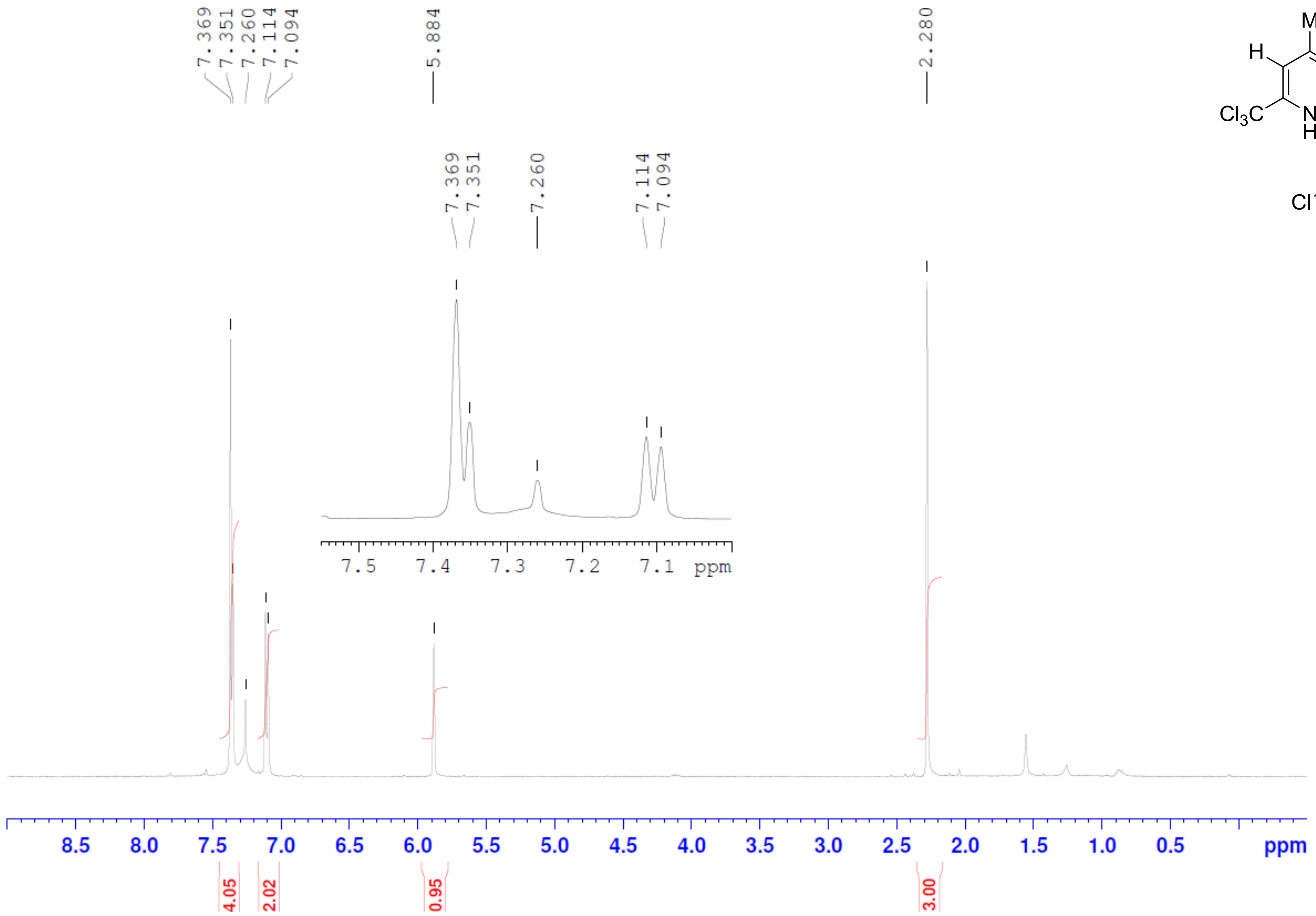

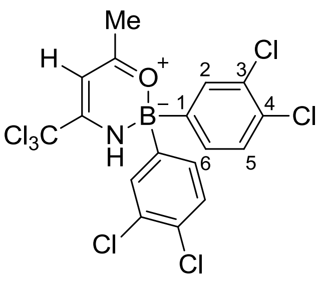

^{1}H NMR spectrum ($CDCl_3$) of 2,2-bis(3,4-dichlorophenyl)-6-methyl-4-(trichloromethyl)-2,3-dihydro-1,3,2-oxazaborinin-1-ium-2-uide (NBC39)

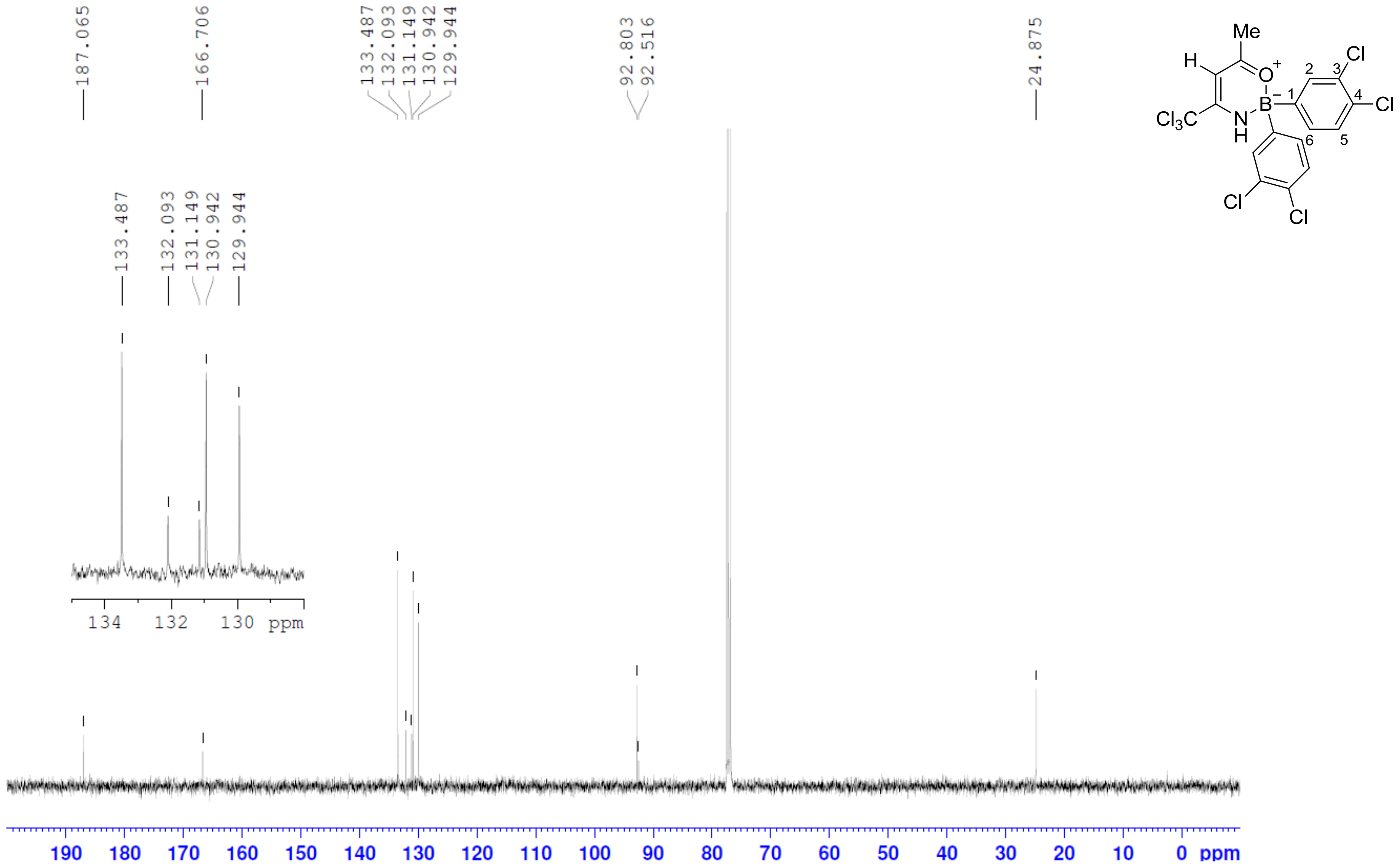

^{13}C NMR spectrum ($CDCl_3$) of 2,2-bis(3,4-dichlorophenyl)-6-methyl-4-(trichloromethyl)-2,3-dihydro-1,3,2-oxazaborinin-1-ium-2-uide (NBC39)

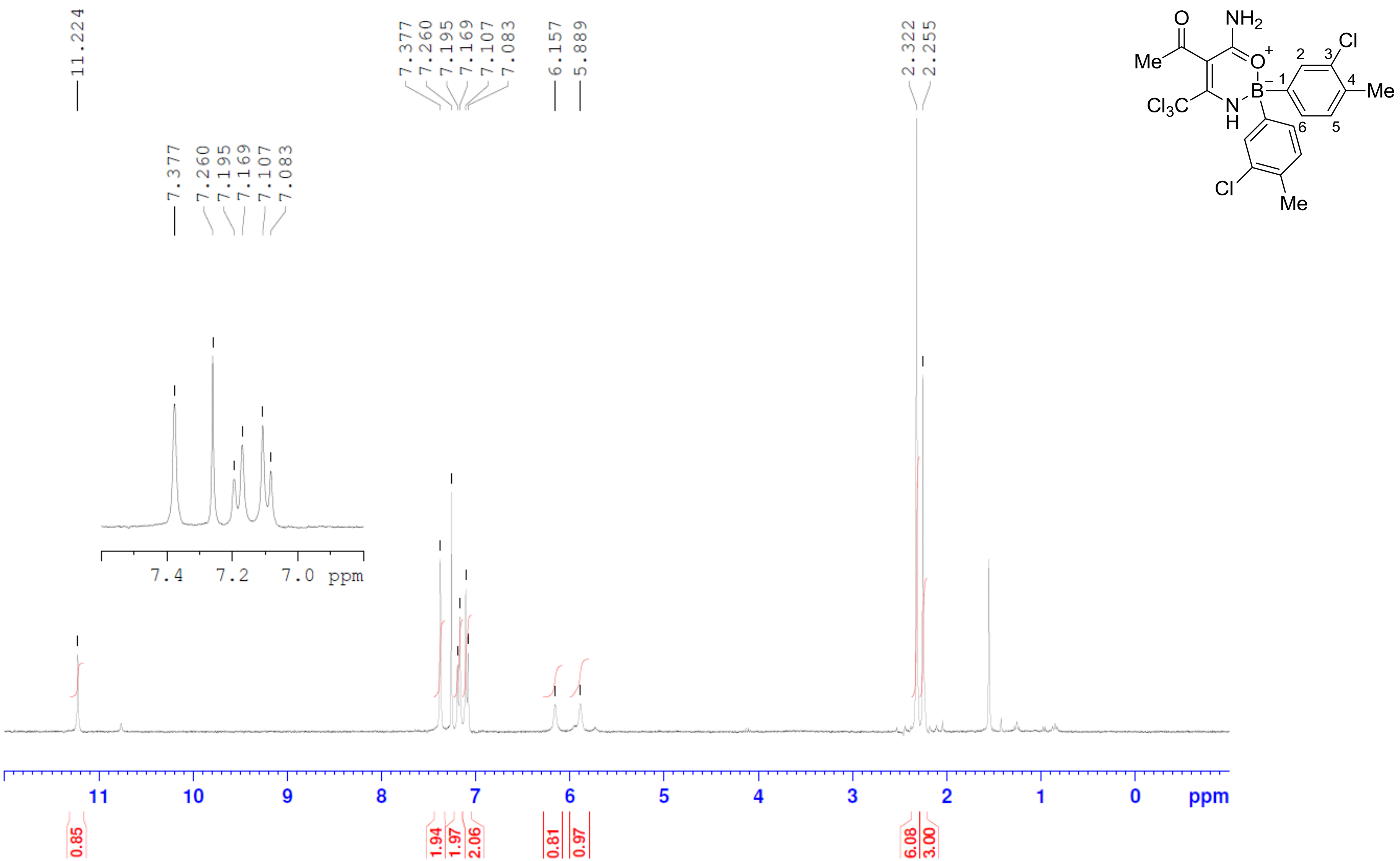

^{1}H NMR spectrum ($CDCl_3$) of 5-acetyl-6-amino-2,2-bis(3-chloro-4-methylphenyl)-4-(trichloromethyl)-2,3-dihydro-1,3,2-oxazaborinin-1-ium-2-uide (NBC40)

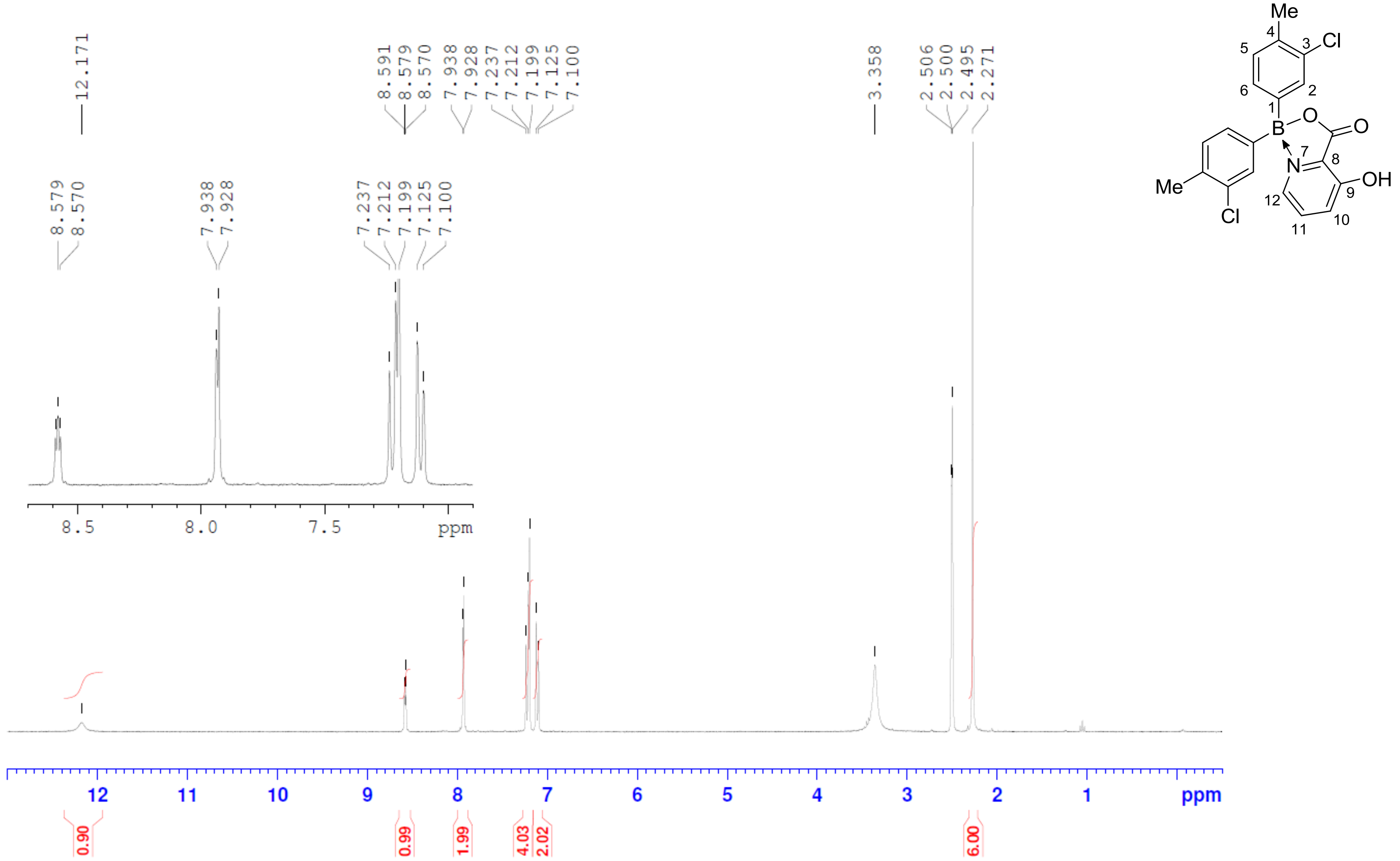

^{1}H NMR spectrum (DMSO-d_6) of 3-hydroxypyridine-2-carbonyloxy-bis(3-chloro-4-methylphenyl) borane (AN0128)

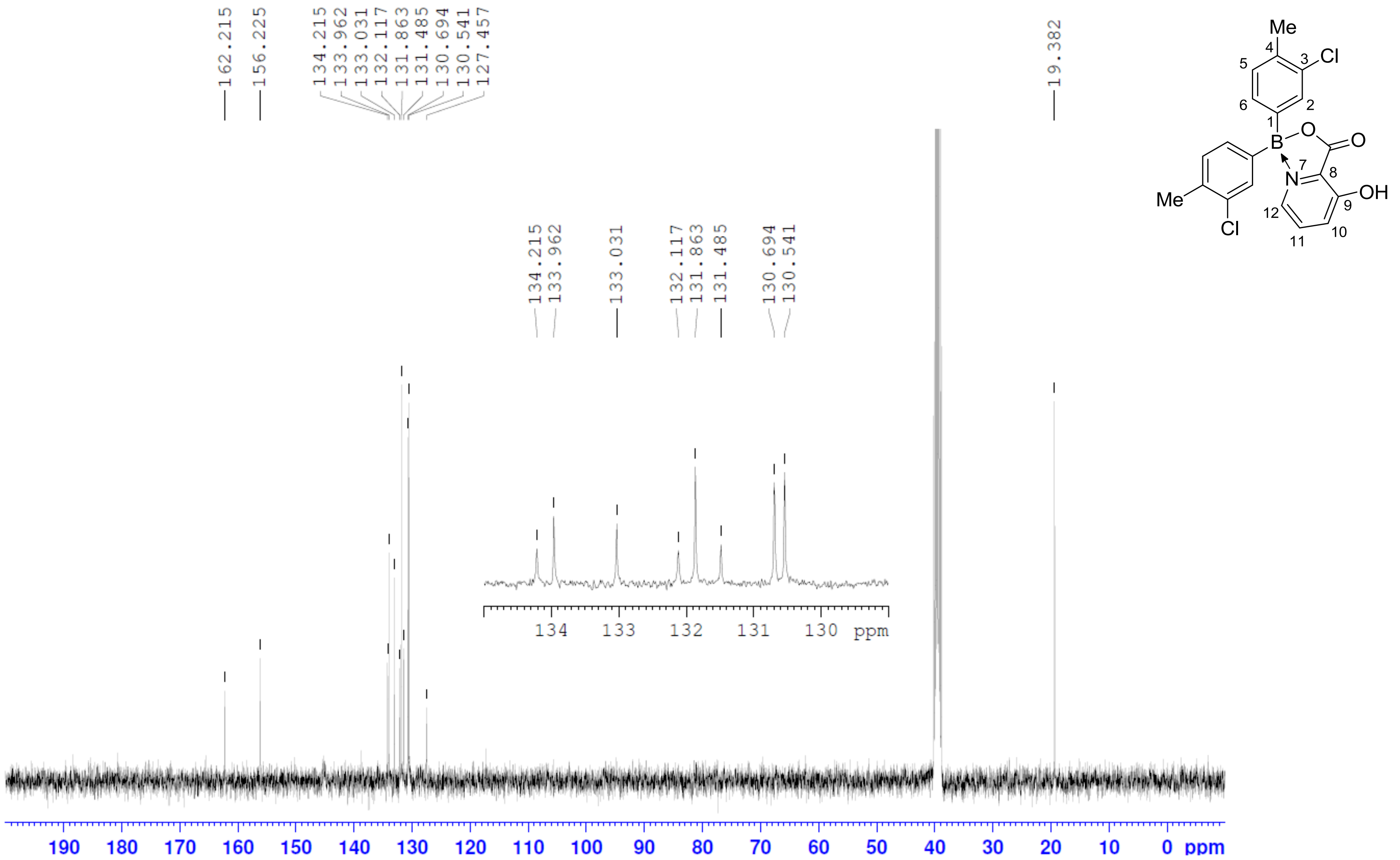

^{13}C NMR spectrum (DMSO-d_6) of 3-hydroxypyridine-2-carbonyloxy-bis(3-chloro-4-methylphenyl) borane (AN0128)

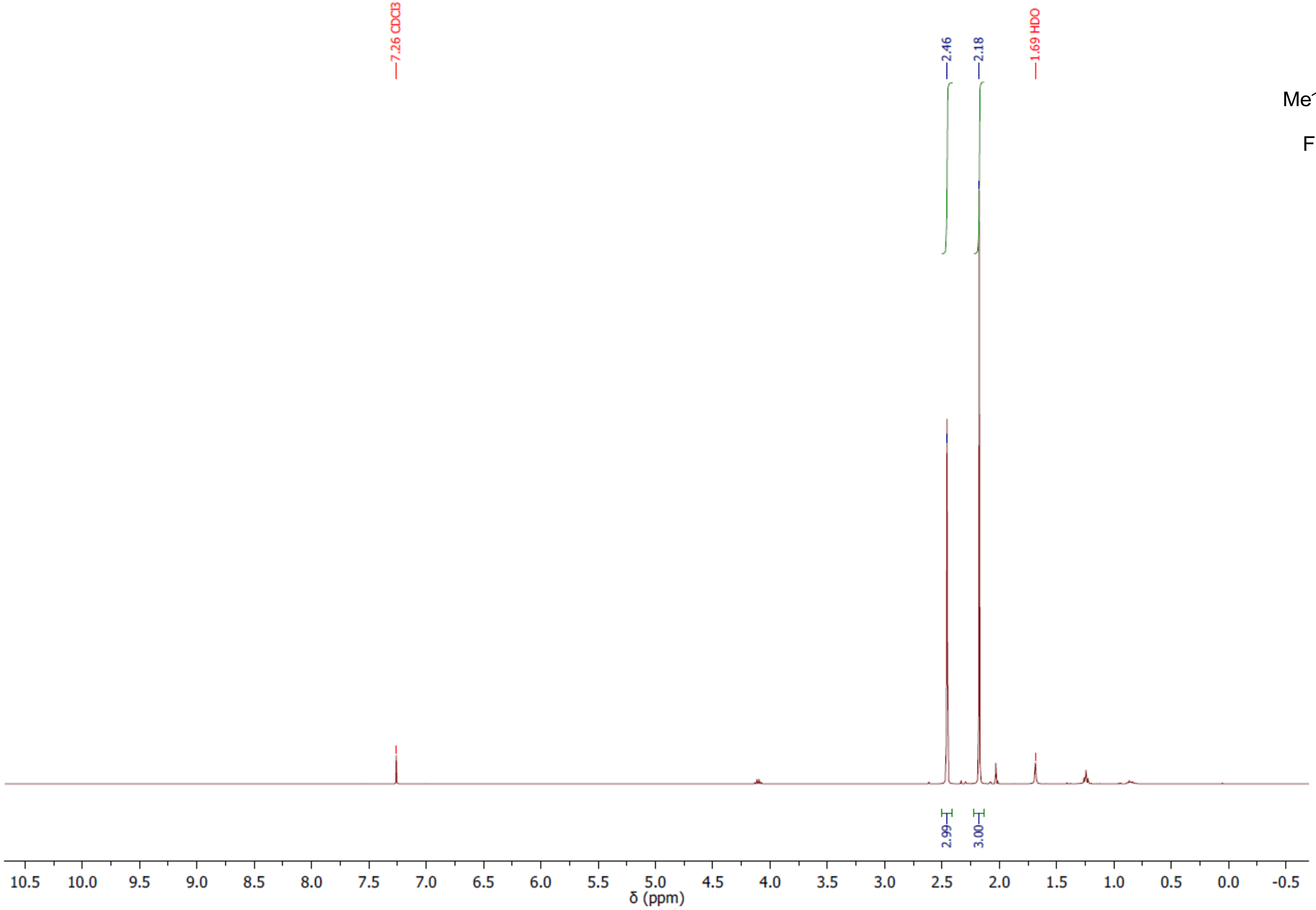

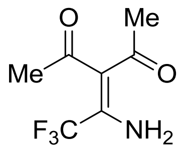

^{1}H NMR spectrum ($CDCl_3$) of 3-(1-amino-2,2,2-trifluoroethylidene)pentane-2,4-dione (**8**)

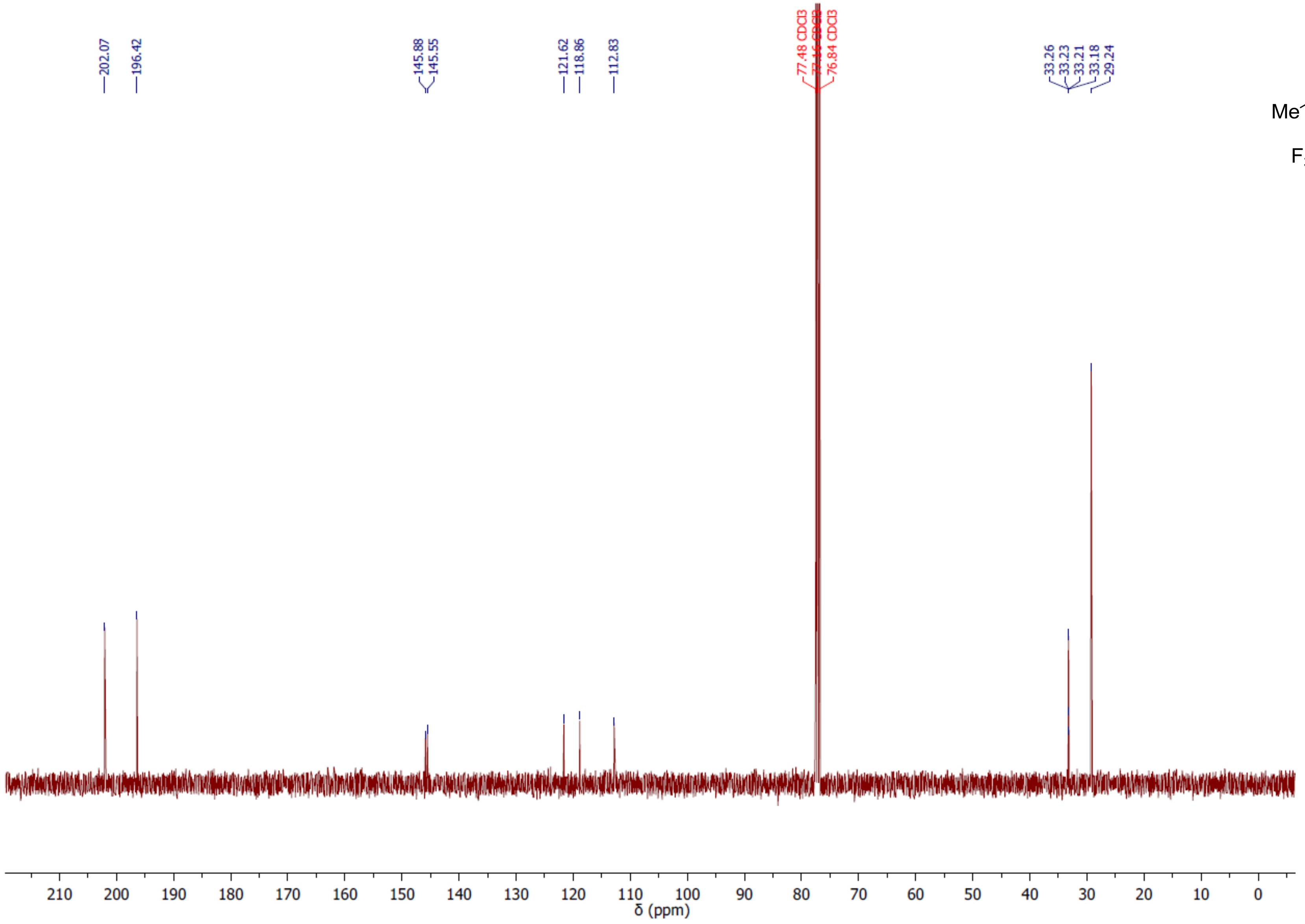

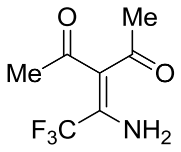

^{13}C NMR spectrum ($CDCl_3$) of 3-(1-amino-2,2,2-trifluoroethylidene)pentane-2,4-dione (**8**)

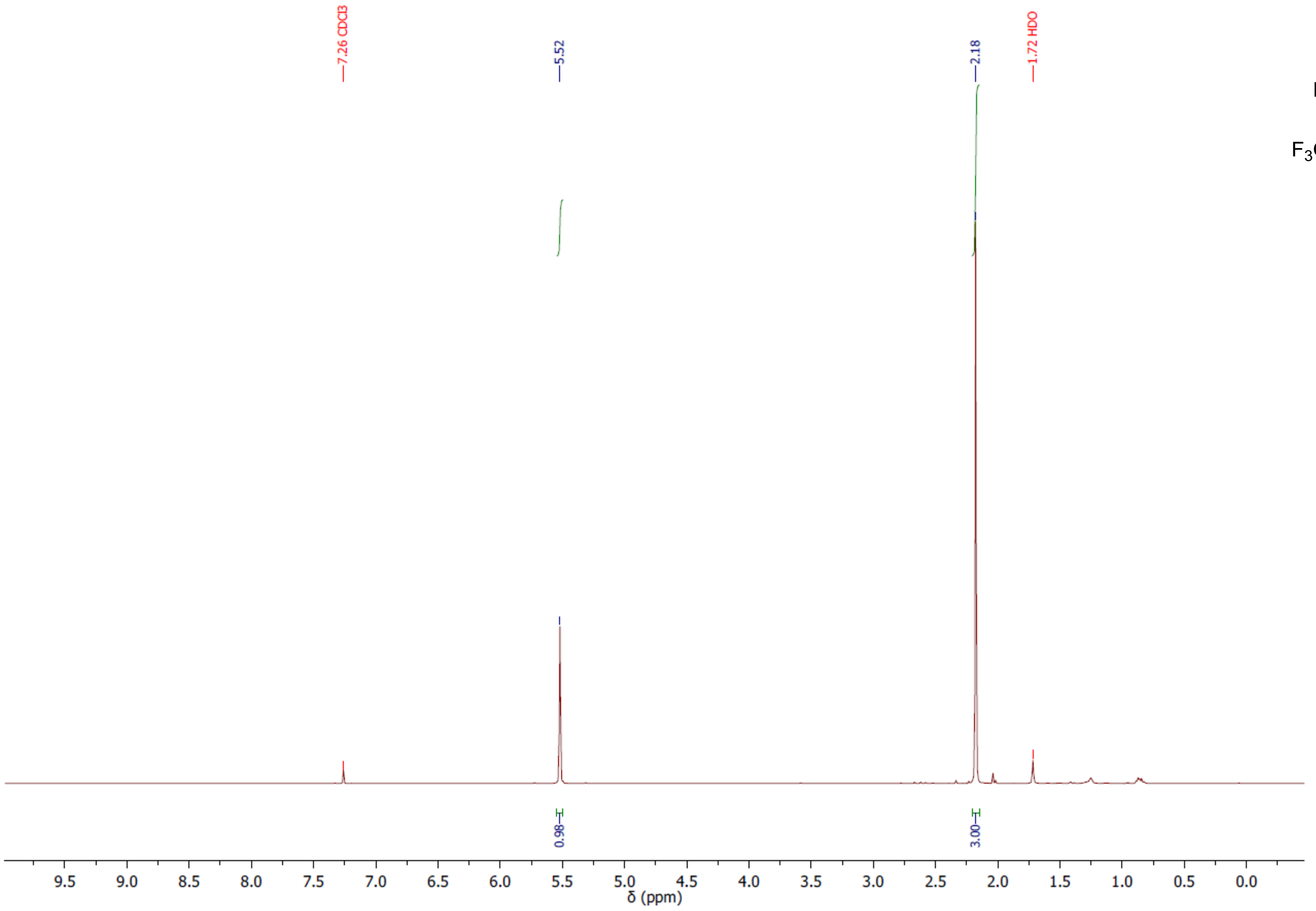

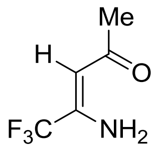

^{1}H NMR spectrum ($CDCl_3$) of (*Z*)-4-amino-5,5,5-trifluoropent-3-en-2-one (**10**)

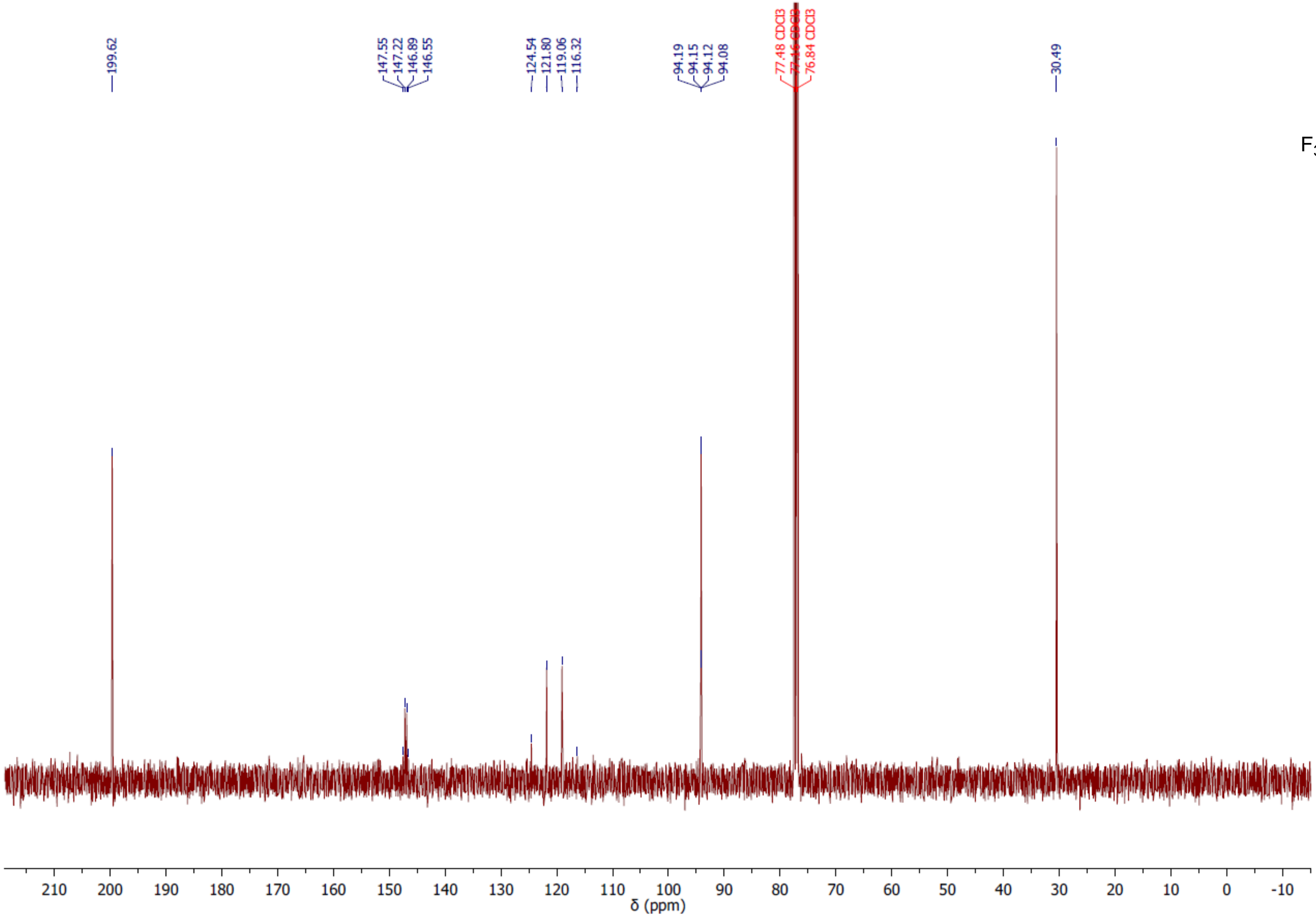

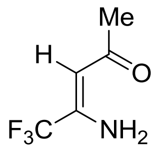

^{13}C NMR spectrum ($CDCl_3$) of (*Z*)-4-amino-5,5,5-trifluoropent-3-en-2-one (**10**)

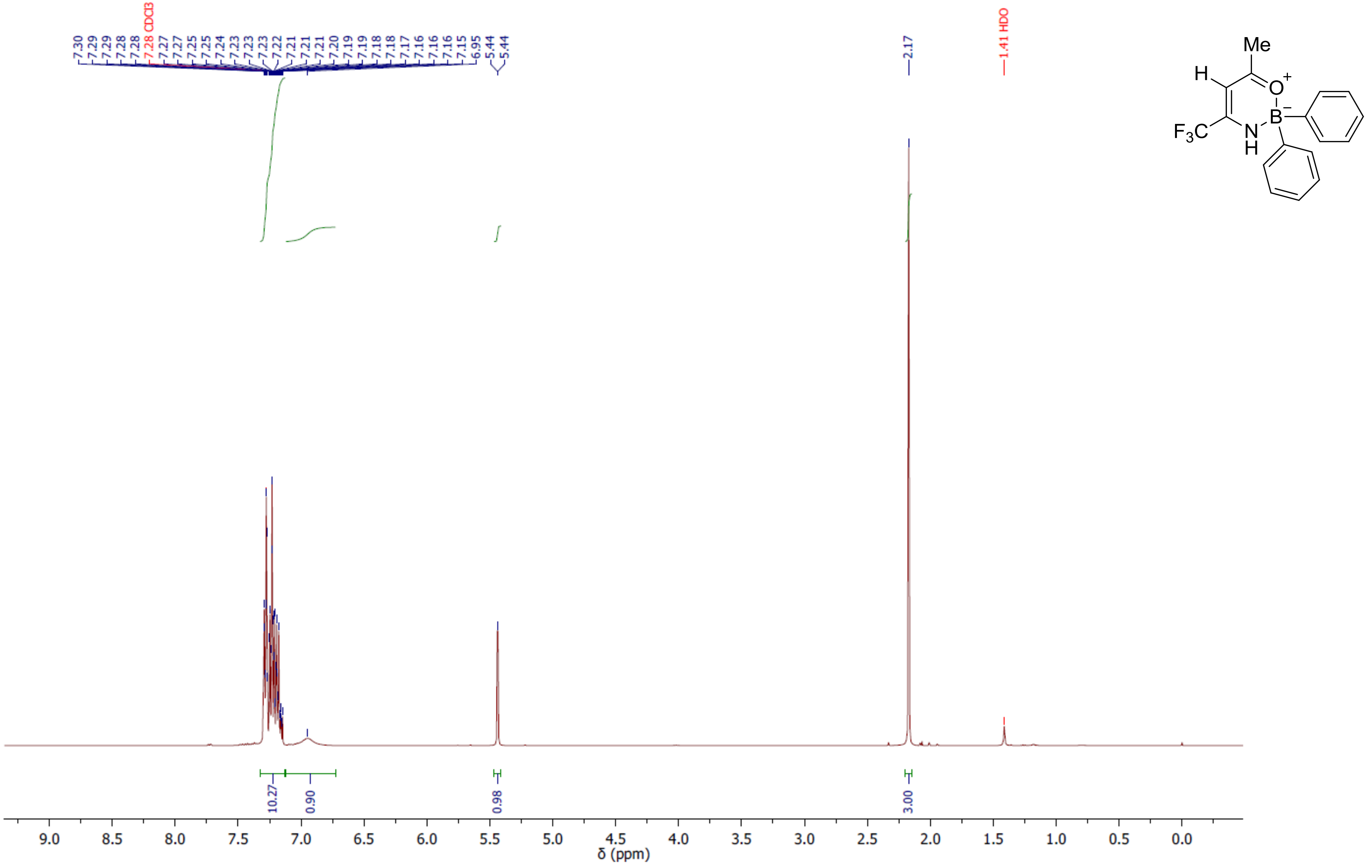

^{1}H NMR spectrum ($CDCl_3$) of 6-methyl-2,2-diphenyl-4-(trifluoromethyl)-2,3-dihydro-1,3,2-oxazaborinin-1-ium-2-uide (NBC41)

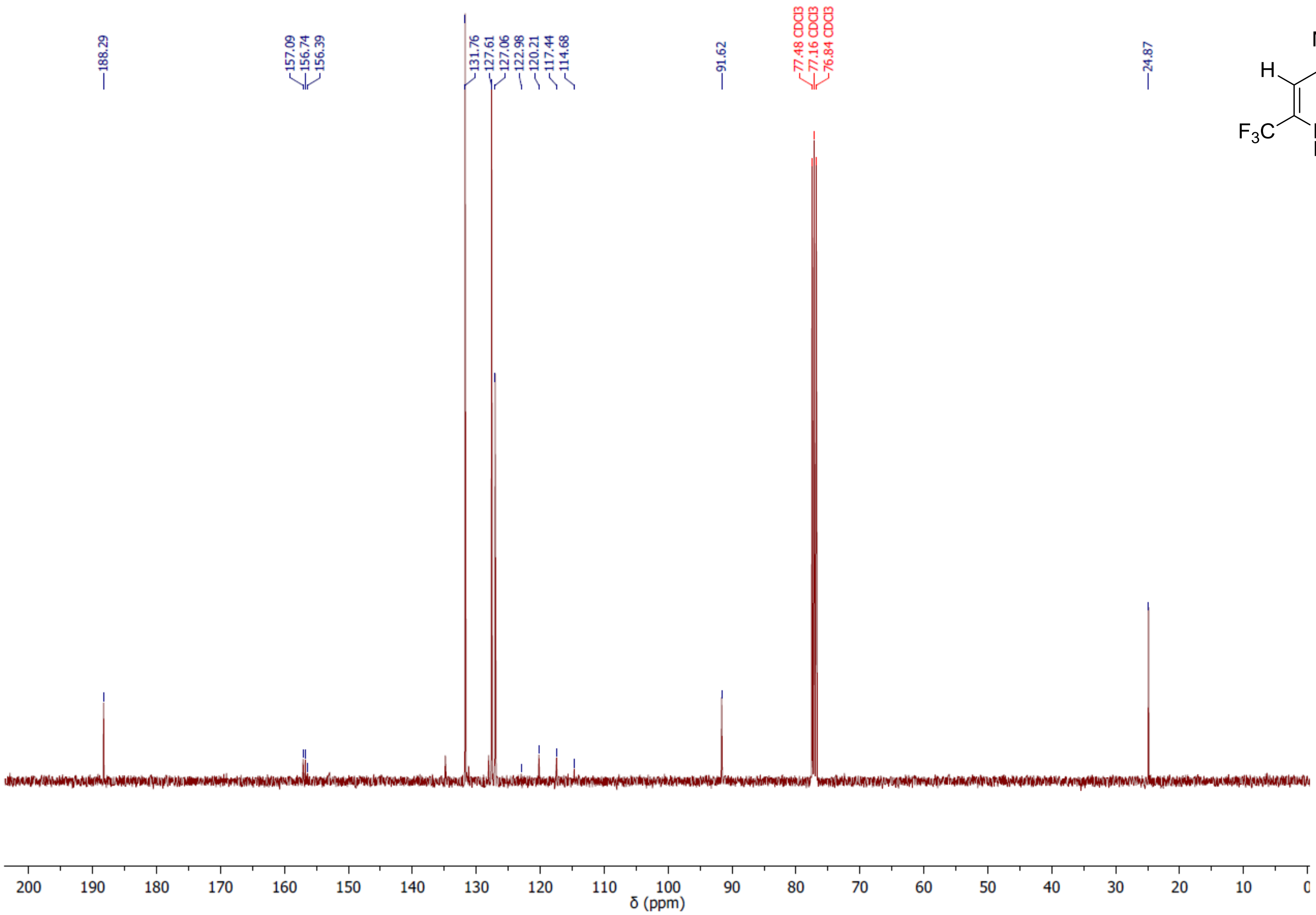

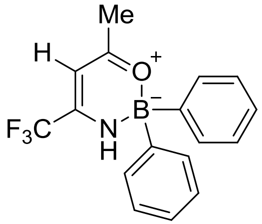

^{13}C NMR spectrum ($CDCl_3$) of 6-methyl-2,2-diphenyl-4-(trifluoromethyl)-2,3-dihydro-1,3,2-oxazaborinin-1-ium-2-uide (NBC41)

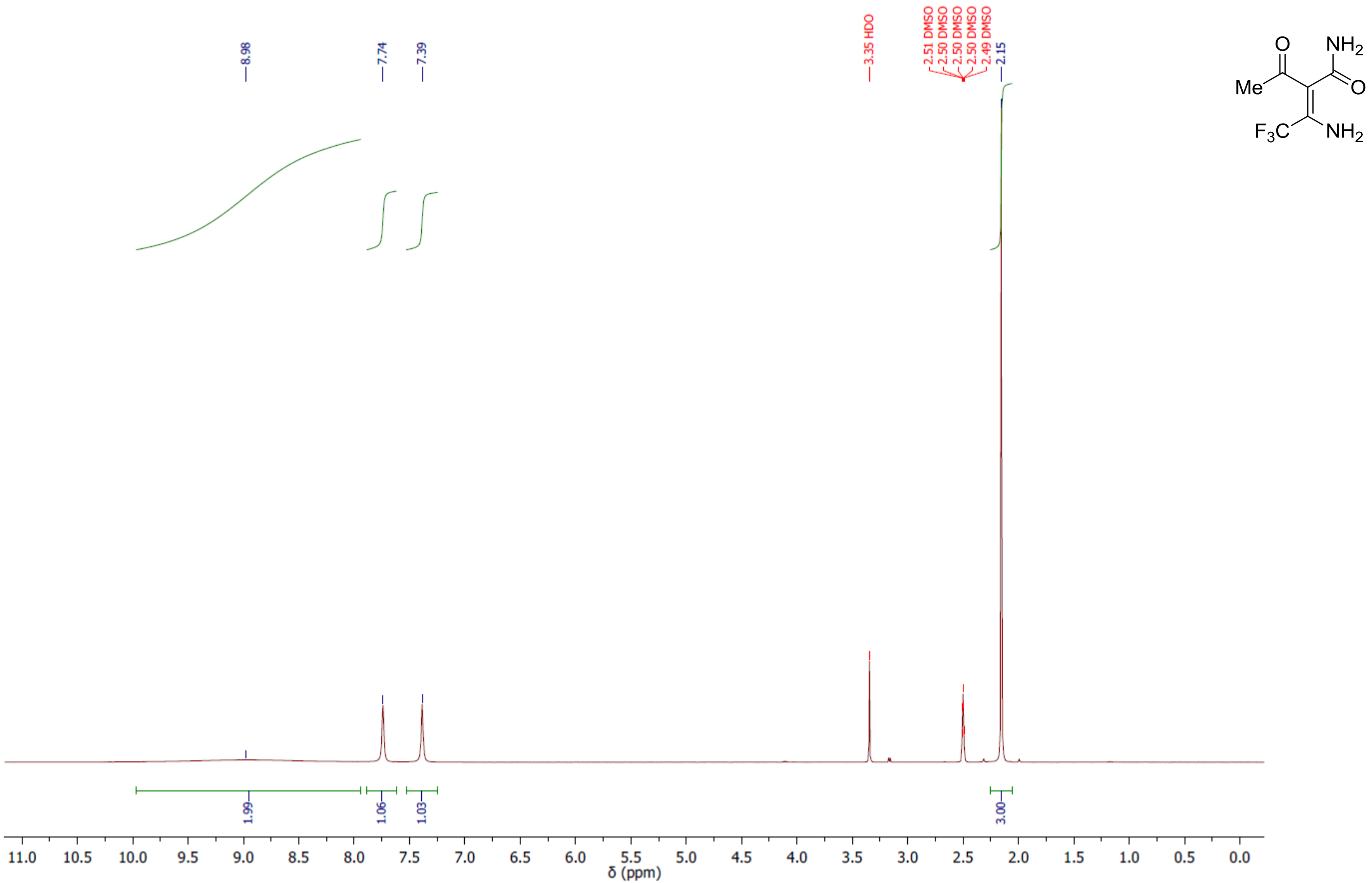

^{1}H NMR spectrum (DMSO-d_6) of (*Z*)-2-acetyl-3-amino-4,4,4-trifluorobut-2-enamide (**9**)

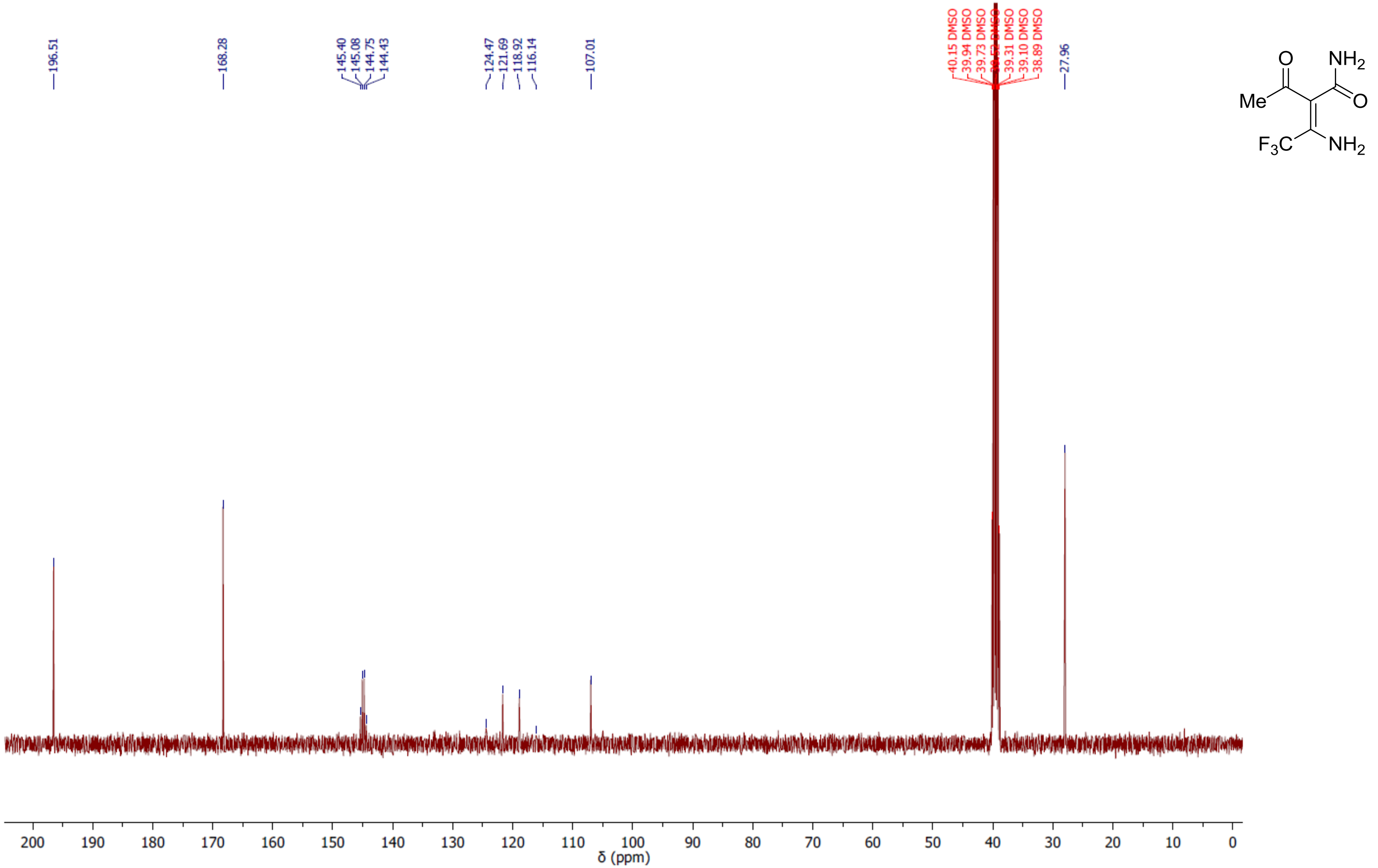

^{13}C NMR spectrum (DMSO-d_6) of (*Z*)-2-acetyl-3-amino-4,4,4-trifluorobut-2-enamide (**9**)

Acquisition Time (sec)	5.3084		
Comment	5 mm PABBO BB-1H/D Z-GRD Z828401/0055		
D	2.4	D1	2.4
DE	6	DS	2
Date	06 Nov 2017 10:36:44		
Date Stamp	06 Nov 2017 10:36:44		
File Name	\\nask.man.ac.uk\home$\Desktop\agb537 freeze stab\10\PDATA\1\1r		
Frequency (MHz)	300.1300	GB	0
INSTRUM	<spect>	LB	0.3
NS	16	Nucleus	1H
Number of Transients	16	Origin	spect
Original Points Count	32768	Owner	nmr
PC	1		
PROBHD	<5 mm PABBO BB-1H/D Z-GRD Z828401/0055 >		
PULPROG	<zg30>	Points Count	32768
Pulse Sequence	zg30	Receiver Gain	1024.00
SF	300.13	SFO1	300.131407219883
SI	32768	SSB	0
SW(cyclical) (Hz)	6172.84	SWH	6172.83950617284
Solvent	CHLOROFORM-d		
Spectrum Offset (Hz)	1404.3987	Spectrum Type	standard
Sweep Width (Hz)	6172.65	TD	65536
TD0	1	TE	295.56
Temperature (degree C)	22.560	UNC1	<1H>
WDW	1		

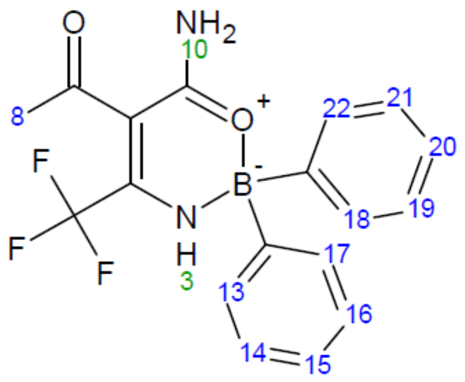

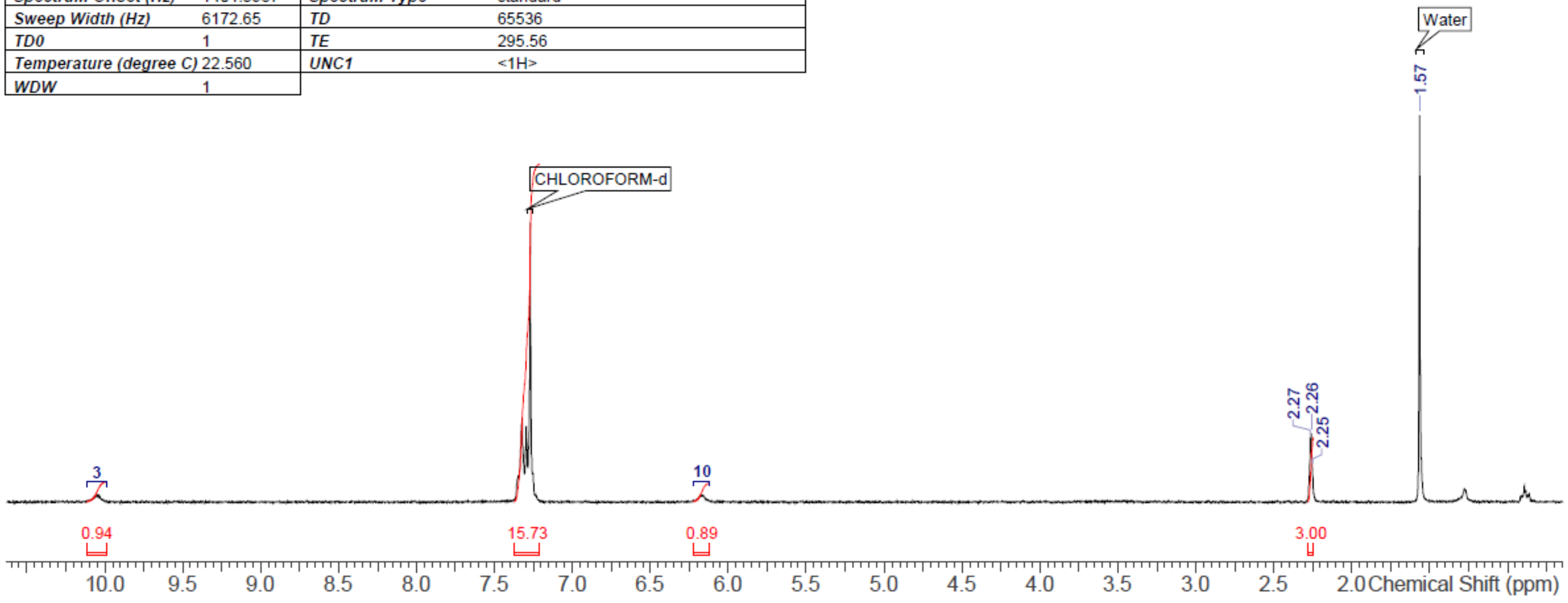

^{1}H NMR spectrum ($CDCl_3$) of 5-acetyl-6-amino-2,2-diphenyl-4-(trifluoromethyl)-2,3-dihydro-1,3,2-oxazaborinin-1-ium-2-uide (NBC42)

Acquisition Time (sec)	1.8219	Comment	James-537
D	0.00345	D1	2
DE	6.5	DS	4
Date	08 Nov 2017 04:50:01		
Date Stamp	08 Nov 2017 04:50:01		
File Name	\\nask.man.ac.uk\home$\Desktop\40\PDATA\1\1r		
Frequency (MHz)	75.4678	GB	0
INSTRUM	<spect>	LB	1
NS	10240	Nucleus	13C
Number of Transients	10240	Origin	spect
Original Points Count	32768	Owner	nmr
PC	1.4		
PROBHD	<5 mm PABBO BB-1H/D Z-GRD Z828401/0055 >		
PULPROG	<zgpg30>	Points Count	32768
Pulse Sequence	zgpg30	Receiver Gain	32768.00
SF	75.467749	SFO1	75.4752953008526
SI	32768	SSB	0
SW(cyclical) (Hz)	17985.61	SWH	17985.6115107914
Solvent	CHLOROFORM-d		
Spectrum Offset (Hz)	7545.1973	Spectrum Type	standard
Sweep Width (Hz)	17985.06	TD	65536
TD0	1	TE	296.56
Temperature (degree C)	23.560	UNC1	<13C>
WDW	1		

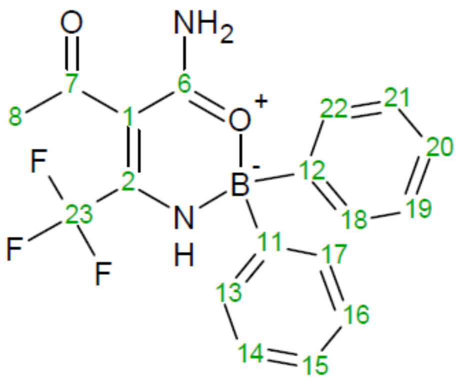

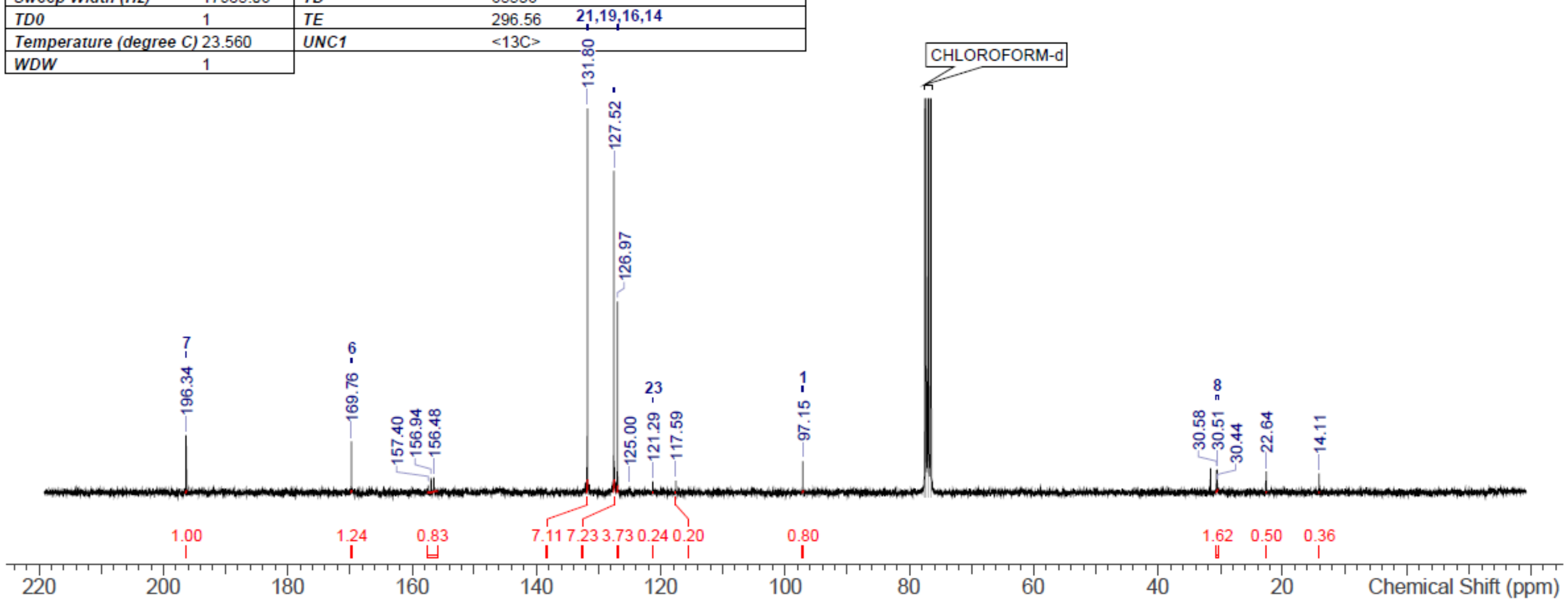

^{13}C NMR spectrum ($CDCl_3$) of 5-acetyl-6-amino-2,2-diphenyl-4-(trifluoromethyl)-2,3-dihydro-1,3,2-oxazaborinin-1-ium-2-uide (NBC42)
